# A Field-Theory Approach for Modeling Dissipative Relativistic Fluids

**DOI:** 10.3390/e26080621

**Published:** 2024-07-24

**Authors:** Nils Andersson, Thomas Celora, Gregory Comer, Ian Hawke

**Affiliations:** 1 Mathematical Sciences and STAG Research Centre, University of Southampton, Southampton SO17 1BJ, UK; na@maths.soton.ac.uk (N.A.); i.hawke@soton.ac.uk (I.H.); 2 Institute of Space Sciences (ICE, CSIC), Campus UAB, Carrer de Magrans, 08193 Barcelona, Spain; celora@ice.csic.es; 3 Department of Physics, Saint Louis University, St. Louis, MO 63156-0907, USA

**Keywords:** relativistic fluids, dissipation, field theory

## Abstract

We develop an action principle for producing a single-fluid two-constituent system with dissipation in general relativity. The two constituents in the model are particles and entropy. The particle flux creation rate is taken to be zero, while the entropy creation rate is non-zero. Building on previous work, it is demonstrated that a new term (the proper time derivative of the matter space “metric”) is required in the Lagrangian in order to produce terms typically associated with bulk and shear viscosity. Equations of motion, entropy creation rate, and energy–momentum–stress tensor are derived. Using an Onsager approach of identifying thermodynamic “forces” and “fluxes”, a model is produced which delivers the same entropy creation rate as the standard, relativistic Navier–Stokes equations. This result is then contrasted with a model generated in the spirit of the action principle, which takes as its starting point a specific Lagrangian and then produces the equations of motion, entropy creation rate, and energy–momentum–stress tensor. Unlike the equations derived from Onsager reasoning, where the analogs of the bulk and shear viscosity coefficients are prescribed “externally”, we find that the forms of the coefficients in the second example are a direct result of the specified Lagrangian. Furthermore, the coefficients are shown to satisfy evolution equations along the fluid worldline, also a product of the specific Lagrangian.

## 1. Introduction

Breakthrough progress in gravitational-wave astronomy prompts us to revisit “old questions” in relativistic fluid dynamics. In order to provide robust models of binary neutron star mergers (like the celebrated GW170817 event [1,2]) and mixed binary systems involving a neutron star and a black hole (like the recently announced GW230529 event [3]), we need to carry out large-scale numerical simulations incorporating as much of the extreme physics as we can manage [4,5]. In addition to the “live” spacetime of Einstein’s gravity, our simulations need to include the complex matter physics that comes into play at densities beyond nuclear saturation. These aspects must be represented faithfully in order to allow reliable parameter extraction from observed signals. Somewhat colloquially, the stated aim is to “constrain the equation of state” of supranuclear density matter. However, this aim includes a number of issues associated with the systematics of simulations and the extracted model waveforms. This, in turn, raises problems which become pressing for the development of the next generation of gravitational-wave instruments (the Einstein Telescope in Europe and Cosmic Explorer in the USA). These instruments will be sensitive at higher frequencies than the current LIGO–Virgo–Kagra interferometers and are expected to observe the post-merger phase, in addition to the late inspiral phase currently seen.

State-of-the-art simulations tell us that binary mergers involve high-density matter at temperatures close to those reached in terrestrial collider experiments (up to 100 MeV) [6]. At these extreme temperatures, the fluid will be far from thermodynamical equilibrium and the role of neutrinos is expected to be paramount [6]. Recent numerical relativity experiments [7,8,9] indicate that out-of-equilibrium physics (in the form of bulk vicosity and/or neutrino transport) will affect the gravitational-wave signal at a “detectable” level. In order to explore the relevant physics, we evidently need to incorporate non-equilibrium aspects in our numerical simulations. In effect, we need to consider dissipative general relativistic fluid dynamics [10].

The implementation of dissipation in relativistic fluid dynamics is known to be tricky, both conceptually and practically. While there has been important recent progress on issues relating to stability and causality [11,12], we still do not have a universally agreed-upon “framework” that would allow us to consider the complete range of physics that comes into play in neutron star mergers. Mergers combine a highly energetic, turbulent flow of beyond-nuclear-density matter; strong magnetic fields; and a dynamical spacetime generating copious amounts of gravitational waves. These events are unique because they operate over an impressive range of spatial scales. At the smallest scales, they provide data for the matter equation of state [13,14,15,16], while at large scales they may form long-lived merger remnants (possibly eventually forming black holes [4,5,17]). Rapid nuclear reactions during low-density matter outflows may lead to observable kilonova signatures [18]. Observed short gamma-ray bursts may be explained as the twisting of the stars’ magnetic field, which would help collimate an emerging jet [19]. Multi-messenger observations of these events will—at some level—encode dissipative aspects (ranging from the bulk viscosity in the merger remnant [6,8] to resistivity affecting the evolution of the magnetic field [20,21,22]).

Arguably, the most “complete” framework for modelling the physics we need to consider is the fully covariant variational approach reviewed in [10]. Notably, recent developments of the variational strategy include dissipative effects [23]. This effort is motivated by the requirements from gravitational-wave astronomy, and provides an action principle for general relativistic multi-fluid systems for which no explicit reference to an equilibrium state is required and as a result the field equations are fully non-linear. This is in sharp contrast to other models for dissipative relativistic fluid dynamics which build on a phenomenological derivative expansion (away from a supposed equilibrium state). The main idea of the variational model is that the dynamical degrees of freedom of fluids are captured by fluxes, and if the flux for a fluid has non-zero covariant divergence, or, equivalently, its associated dual three-form is not closed, then there will be dissipation. Conceptually, the idea is clear but we are still quite far from turning this understanding into a complete “workable” model.

The aim of the present discussion is to take steps to improve the situation by building an explicit action principle which connects with the familiar Navier–Stokes equations. The spirit of the approach is very similar to that of, for example, refs. [24,25,26], where the matter space coordinates are treated as fields in the variation. The close connection between the two approaches has already been discussed in detail in [10]. While we will not make further comparison here, it is clear that progress in either direction can be translated into the other framework and it would certainly be worth paying attention to this in the future. Our focus here is on the geometrical aspects of the problem—including relevant symmetries—some of which are directly connected with the spacetime metric and hence unique to the context of general relativity. We focus on the mathematical formalism, leaving a discussion of the deeper connection to statistical mechanics and the precise role of microscopic fluctuations (see, e.g., ref. [27] for a useful survey) for follow-up work. This strategy makes sense because issues related to the underlying physics are somewhat distinct from the geometric aspects which are the focus here. The deeper connections need to be explored once a self-consistent mathematical framework is in place so work in this direction should certainly be encouraged.

We could perhaps claim to be motivated by the old (often paraphased) proverb that necessity is the mother of invention. Google suggests that one of the earliest statements of this proverb is to be found in the Aesop’s Fable “The Crow and the Pitcher” (see, e.g., https://read.gov/aesop/001.html (accessed on 30 May 2024).) Alternatively, we can draw inspiration from Plato’s *Republic* and the comment “our need will be the real creator” (Benjamin Jowett, *Plato’s Republic: The Greek Text*, 1894, 3:82 “Notes” Jowett, Book II, 369c). Staying closer to science, Alfred North Whitehead argued in an address to the Mathematical Association of England that “the basis of invention is science, and science is almost wholly the outgrowth of pleasurable intellectual curiosity”. Perhaps curiosity—pleasurable or not—is the main driver for the current effort? Maybe we are just stumbling around in the dark, with “necessity is the mother of futile dodges” (Julius A. Sigler, *Education: Ends and Means*. University Press of America. p. 140.) in mind… There are different possible attitudes, but theoretical, experimental, and observational investigations of viscous fluids have, at some time or other, embodied the sentiments of each of the above quotes. This may simply be a reflection of how challenging the problem is. The work presented here provides, we believe, a unique perspective (but we cannot yet say if this is more than a futile dodge).

Our discussion will introduce a number of “simplifications”. Most notably, we will restrict ourselves to a single-fluid model. In some sense, this is against “better judgement” because we know that issues like heat/entropy flow require a multi-fluid treatment [10]. Moreover, the variational framework readily allows for multi-fluid aspects to be incorporated. However, if we want to make contact with numerical simulations (and we do!) then it must be noted that such efforts reduce the analysis to a single fluid whenever this is possible. Hence, it makes sense to see how far we can go if we restrict the variational discussion in this sense from the outset. The obvious caveat to this statement of intent is that we should perhaps not expect the effort to be completely successful. We are cutting corners and this ought to impact on the model we arrive at. Having said that, we expect to learn useful lessons from the exercise. The calculation we present is perhaps mainly interesting from a conceptual perspective, but the derivation also highlights aspects that need to be included in more realistic models. For example, we will show that a new fluid variable (the proper time derivative of the matter space “metric”) must be included in the original Lagrangian of [23] in order to recover the expected terms associated with bulk and shear viscosity. This new inclusion, in turn, affects the field equations, the entropy creation rate, and the energy–momentum–stress tensor. Additionally, we provide an explicit formulation of the matter space entropy three-form, going beyond the phenomenology explored in previous work. The results show that evolution equations along worldlines naturally arise in the model, as one might expect from a relativistic formulation.

In Section 2, the generic action is written down and a variation with respect to the field variables (particle and entropy flux and the spacetime metric) is given. In Section 3, abstract, three-dimensional “matter” spaces are introduced so that the fluxes can be reformulated in such a way that the action principle becomes viable. Section 4 uses the same approach as [23] to build the required variations of the field variables, in particular, the Lagrangian displacement in Section 4.2. While the approach is the same, derivatives of the matter space metrics are assumed in the generic functional form of the action. This is because models like thetraditional Navier–Stokes are not possible without such derivatives in the Lagrangian. In Section 5, all the ingredients are stirred together and poured back into the initial variation of Section 2. The fluid field equation, entropy creation rate, and energy–momentum–stress tensor are derived. In Section 6, a specific form for the Lagrangian is written down. In Appendix A, we provide details of the derivations of key elements of the formalism. While the results of the derivations are essential to delivering the final product, the calculations themselves are not necessary during a first reading of this paper.

## 2. The Fluid Action

In the variational approach, the equations of motion are derived from an action principle which has as its Lagrangian the so-called “master” function Λ (see [10] for an extensive review). For a finite-temperature single-component system (as considered here), the master function is a function of all the independent scalars which can be built using the spacetime metric $g_{ab}$, the particle flux $n^{a}$, and the entropy flux $s^{a}$. However, here we restrict ourselves by only considering $n^{2}=-g_{ab}n^{a}n^{b}$ and $s^{2}=-g_{ab}s^{a}s^{b}$ (excluding the quantity $g_{ab}n^{a}s^{b}$, known to be associated with entropy entrainment [10]). The action is then given by

(1) $$S_{F}=\int_{M}d^{4}x\sqrt{-g}\Lambda \left(n^{2},s^{2}\right)\;.$$

The variation of $S_{F}$ with respect to $n^{a}$, $s^{a}$, and $g_{ab}$ is

(2) $$\begin{array}{cc} \delta S_{F} & =\int_{M}d^{4}x\delta \left(\sqrt{-g}\Lambda \right)\\ \; & =\int_{M}d^{4}x\sqrt{-g}\left[\mu_{a}\delta n^{a}+\Theta_{a}\delta s^{a}+\frac{1}{2}\left(\Lambda g^{ab}+\mu^{a}n^{b}+\Theta^{a}s^{b}\right)\delta g_{ab}\right]\;, \end{array}$$

where we have used the fact that

(3) $$\delta \sqrt{-g}=\frac{1}{2}\sqrt{-g}g^{ab}\delta g_{ab}$$

and defined

(4) $$\begin{array}{cc} \mu_{a} & =-2\frac{\partial \Lambda }{\partial n^{2}}n_{a}\;, \end{array}$$

(5) $$\begin{array}{cc} \Theta_{a} & =-2\frac{\partial \Lambda }{\partial s^{2}}s_{a}\;. \end{array}$$

As we restrict our analysis to systems with a single-fluid degree of freedom, the two constituents, particles and entropy, must be co-moving. We denote the corresponding unit four-velocity as $u^{a}$, with normalization $u_{a}u^{a}=-1$ (in geometric units). The particle flux is now $n^{a}=nu^{a}$, and the entropy flux is $s^{a}=su^{a}$, where the particle density is given by $n=-u_{a}n^{a}$ and the entropy density is $s=-u_{a}s^{a}$. We also note that the chemical potential is given by $\mu =-u^{a}\mu_{a}$ and the temperature follows from $T=-u^{a}\Theta_{a}$.

The derivation of the equations of motion is complicated by the fact that our variation of the fluxes $\delta n^{a}$ and $\delta s^{a}$ must involve, indirectly, the variation of the worldlines given by $u^{a}$. Because $u_{a}u^{a}=-1$ everywhere, it has only three degrees of freedom. The impact of this can be seen already in $\delta S_{F}$ above. The equations of motion result when arbitrary variations of the field degrees of freedom do not change $S_{F}$ to linear order; i.e., $\delta S_{F}=0$. If we consider arbitrary variations $\delta n^{a}$ and $\delta s^{a}$, then the equations of motion are simply $\mu_{a}=\Theta_{a}=0$, which do not recover the simplest perfect fluid equations.

As shown in [28], building a viable action for two different “particle” constituents, such as matter and entropy, and one four-velocity, is straight-forward in the non-dissipative (perfect fluid) regime; even the generalization to a non-dissipative system of, say, *M*-constituents and *N*-fluids follows naturally (see [10] for details). Building on this, Andersson and Comer [23] demonstrated how to take the basic principles built into these actions and developed a fully non-linear set of field equations for dissipative fluids. But, as we will demonstrate in the next section, it is not straight-forward, a priori, to extend single-fluid actions to dissipative systems (as represented by, for example, the traditional Navier–Stokes equations).

## 3. Matter Space and Flux Setup

Let us introduce the necessary ingredients of a viable action principle for a single fluid of matter and entropy which has dissipation. The first step is to introduce two abstract three-dimensional Riemannian (“matter space”) manifolds, whose individual points correspond to individual fluid worldlines in spacetime. The second step is to assume that the two manifolds are diffeomorphic to each other. A fair bit of infrastructure will have to be built before reaching the action principle and the resulting field equations; in particular, a lot of detail on the so-called matter space metrics must be included as these were shown in [23] to be essential elements required for dissipation. Some of the more tedious details of the infrastructure construction are presented in Appendix A.

### 3.1. The Matter Space Setups

First of all, we introduce the two three-dimensional Riemannian manifolds that are diffeomorphic to each other. The first of these, the abstract particle space, is labeled by the coordinates $X^{A}$ (A=1,2,3), and the second, for the abstract entropy space, is labeled by the coordinates ${\bar{X}}^{A}$. Because the two spaces are diffeomorphic to each other, there are two mappings $f^{A}$ and ${\bar{f}}^{A}$ whereby

(6) $${\bar{X}}^{A}={\bar{f}}^{A}\left(X^{B}\right)\;,\;X^{A}=f^{A}\left({\bar{X}}^{B}\right)\;,$$

and

(7) $$M_{B}^{A}=\frac{\partial f^{A}}{\partial {\bar{X}}^{B}}\;,\;{\bar{M}}_{B}^{A}=\frac{\partial {\bar{f}}^{A}}{\partial X^{B}}\;∋\;M_{C}^{A}{\bar{M}}_{B}^{C}={\bar{M}}_{C}^{A}M_{B}^{C}=\delta_{B}^{A}\;.$$

Both sets, $X^{A}$ and ${\bar{X}}^{A}$, are scalar functions on spacetime, with the property that each unique wordline of the field $u^{a}$ is mapped to a unique point $X^{A}$ in the matter space and a unique point ${\bar{X}}^{A}$ in the entropy space.

Next, spacetime-index-carrying objects, like $n^{a}$, $s^{a}$, and the metric $g^{ab}$, can be identified with objects carrying matter space indices (such as the particle and entropy, respectively, densities $n_{ABC}$ and $s_{ABC}$ introduced below) through use of the maps

(8) $$\Psi_{a}^{A}=\nabla_{a}X^{A}$$

and

(9) $${\bar{\Psi }}_{a}^{A}=\nabla_{a}{\bar{X}}^{A}\;.$$

The maps are connected to each other via the chain rule, i.e.,

(10) $$\Psi_{a}^{A}=\frac{\partial f^{A}}{\partial {\bar{X}}^{B}}\nabla_{a}{\bar{X}}^{B}=M_{B}^{A}{\bar{\Psi }}_{a}^{B}\;,\;{\bar{\Psi }}_{a}^{A}=\frac{\partial {\bar{f}}^{A}}{\partial X^{B}}\nabla_{a}X^{B}={\bar{M}}_{B}^{A}\Psi_{a}^{B}\;.$$

The four maps $\left\{\Psi_{a}^{A},{\bar{\Psi }}_{a}^{A},{\bar{M}}_{B}^{A},M_{B}^{A}\right\}$ will be shown later to be preserved along the worldlines of $u^{a}$ (i.e., they are Lie-dragged by the fluid flow).

### 3.2. The Particle and Entropy Flux, Chemical Potential, Temperature, and Metric Constructs

The $\Psi_{a}^{A}$ and ${\bar{\Psi }}_{a}^{A}$ maps allow us to “pull-back/push-forward” index-carrying objects in spacetime and the matter spaces. To begin, we replace the fluxes $n^{a}$ and $s^{a}$ with their respective dual three-forms $n_{abc}$ and $s_{abc}$, namely,

(11) $$\begin{array}{c} n_{abc}=\epsilon_{dabc}n^{d}\;,\;n^{a}=\frac{1}{3!}\epsilon^{bcda}n_{bcd}\;,\\ s_{abc}=\epsilon_{dabc}s^{d}\;,\;s^{a}=\frac{1}{3!}\epsilon^{bcda}s_{bcd}\;. \end{array}$$

The particle space three-form $n_{ABC}$ and the entropy space three-form $s_{ABC}$ are then related to the above as

(12) $$n_{abc}=\Psi_{a}^{A}\Psi_{b}^{B}\Psi_{c}^{C}n_{ABC}\;,\;s_{abc}={\bar{\Psi }}_{a}^{A}{\bar{\Psi }}_{b}^{B}{\bar{\Psi }}_{c}^{C}s_{ABC}\;.$$

Similarly, we introduce the dual three-forms for $\mu_{a}$ and $\Theta_{a}$, i.e.

(13) $$\begin{array}{cc} \mu^{abc}=\epsilon^{dabc}\mu_{d}, & \mu_{a}=\frac{1}{3!}\epsilon_{bcda}\mu^{bcd}\;,\\ \Theta^{abc}=\epsilon^{dabc}\Theta_{d}, & \Theta_{a}=\frac{1}{3!}\epsilon_{bcda}\Theta^{bcd}\;, \end{array}$$

to obtain the matter space chemical potential and temperature three-forms, respectively:

(14) $$\mu^{ABC}=\Psi_{a}^{A}\Psi_{b}^{B}\Psi_{c}^{C}\mu^{abc}\;,\;\Theta^{ABC}={\bar{\Psi }}_{a}^{A}{\bar{\Psi }}_{b}^{B}{\bar{\Psi }}_{c}^{C}\Theta^{abc}\;.$$

The remaining dynamical field is the spacetime metric $g^{ab}$. Using the maps $\Psi_{a}^{A}$ and ${\bar{\Psi }}_{a}^{A}$ we may construct three matter space “metrics” $g^{AB}$, ${\bar{g}}^{AB}$, and ${\hat{g}}^{AB}$ (we will see later that these fields are essential components of an action-based dissipative system):

(15) $$\begin{array}{cc} g^{AB} & =\Psi_{a}^{A}\Psi_{b}^{B}g^{ab}=g^{BA}\;, \end{array}$$

(16) $$\begin{array}{cc} {\bar{g}}^{AB} & ={\bar{\Psi }}_{a}^{A}{\bar{\Psi }}_{b}^{B}g^{ab}={\bar{g}}^{BA}\;, \end{array}$$

(17) $$\begin{array}{cc} {\hat{g}}^{AB} & =\Psi_{a}^{A}{\bar{\Psi }}_{b}^{B}g^{ab}={\bar{\Psi }}_{b}^{B}\Psi_{a}^{A}g^{ba}={\hat{g}}^{BA}\;. \end{array}$$

Because of the chain rule, we have

(18) $$\begin{array}{c} g^{AB}=M_{C}^{A}M_{D}^{B}{\bar{\Psi }}_{a}^{C}{\bar{\Psi }}_{b}^{D}g^{ab}=M_{C}^{A}M_{D}^{B}{\bar{g}}^{CD}\;,\\ {\bar{g}}^{AB}={\bar{M}}_{C}^{A}{\bar{M}}_{D}^{B}\Psi_{a}^{C}\Psi_{b}^{D}g^{ab}={\bar{M}}_{C}^{A}{\bar{M}}_{D}^{B}g^{CD}\;,\\ {\hat{g}}^{AB}=\Psi_{a}^{A}{\bar{M}}_{C}^{B}\Psi_{b}^{C}g^{ab}={\bar{M}}_{C}^{B}g^{AC}={\bar{M}}_{C}^{B}g^{CA}\;. \end{array}$$

Locally (on matter space), these objects transform as tensors. However, for our purposes it is better to view the index-carrying objects as matrices and the transformations as matrix products. Note that the use of multiple matter space metrics (although on different, but linked, manifolds) was the way that [23] introduced dissipation into a relativistic action principle.

### 3.3. Mapping $g^{AB}$, ${\bar{g}}^{AB}$, and ${\hat{g}}^{AB}$ to Spacetime Three-Metrics Perpendicular to $u^{a}$

Our goal here is to introduce dissipation into the relativistic fluid theory. It is well established in the literature that the form $\nabla^{a}u^{b}$ is the principal object that shows up in the different channels of dissipation (bulk, shear, etc.). The various channels of dissipation are extracted through the use of a well-known decomposition of $\nabla^{a}u^{b}$, namely,

(19) $$\begin{array}{cc} \nabla^{a}u^{b} & =\sigma^{ab}+\frac{1}{3}\Theta h^{ab}+\varpi^{ab}-u^{a}a^{b}\;,\\ \sigma^{ab} & =\frac{1}{2}\left(h^{ac}\nabla_{c}u^{b}+h^{bc}\nabla_{c}u^{a}\right)-\frac{1}{3}\Theta h^{ab}=\sigma^{ba}\;,\\ \varpi^{ab} & =\frac{1}{2}\left(h^{ac}\nabla_{c}u^{b}-h^{bc}\nabla_{c}u^{a}\right)=-\varpi^{ba}\;,\\ h^{ab} & =g^{ab}+u^{a}u^{b}=h^{ba}\;,\\ a^{a} & =u^{b}\nabla_{b}u^{a}\;,\\ \Theta & =\nabla_{a}u^{a}\;. \end{array}$$

The $h_{ab}$ here is directly connected with $g_{AB}$ since

(20) $$\begin{array}{cc} \Psi_{a}^{A}\Psi_{b}^{B}g_{AB} & =h_{ab}\;. \end{array}$$

Obviously, $h^{ab}u_{b}=h^{ba}u_{b}=0$, which means $\sigma^{ab}u_{b}=\sigma^{ba}u_{b}=0$ and $\varpi^{ab}u_{b}=-\varpi^{ba}u_{b}=0$, as well. It is also the case that $h^{ab}\sigma_{ab}=0$ and $h^{ab}\varpi_{ab}=0$. Finally, because $u_{a}u^{a}=-1$, we have $u^{a}a_{a}=0$.

The pull-back of $g_{AB}$, ${\bar{g}}_{AB}$, and ${\hat{g}}_{AB}$ leads to five distinct “metric” tensors on spacetime which are spacelike with respect to the $u^{a}$ worldlines:

(21) $$\begin{array}{cc} h_{ab} & =\Psi_{a}^{A}\Psi_{b}^{B}g_{AB}={\bar{\Psi }}_{a}^{A}{\bar{\Psi }}_{b}^{B}{\bar{g}}_{AB}=\Psi_{a}^{A}{\bar{\Psi }}_{b}^{B}{\hat{g}}_{AB}={\bar{\Psi }}_{a}^{A}\Psi_{b}^{B}{\hat{g}}_{BA}\;,\\ {\bar{h}}_{ab}^{\left(1\right)} & ={\bar{\Psi }}_{a}^{A}{\bar{\Psi }}_{b}^{B}g_{AB}\;,\;{\bar{h}}_{ab}^{\left(2\right)}=\Psi_{a}^{A}\Psi_{b}^{B}{\bar{g}}_{AB}\;,\\ {\hat{h}}_{ab}^{\left(1\right)} & =\Psi_{a}^{A}{\bar{\Psi }}_{b}^{B}g_{AB}={\bar{\Psi }}_{a}^{B}{\bar{\Psi }}_{b}^{A}{\hat{g}}_{AB}\;,\;{\hat{h}}_{ab}^{\left(2\right)}=\Psi_{a}^{A}\Psi_{b}^{B}{\hat{g}}_{AB}={\bar{\Psi }}_{a}^{A}\Psi_{b}^{B}{\bar{g}}_{AB}\;. \end{array}$$

However, because $h_{ab}u^{b}={\bar{h}}_{ab}^{\left(1\right)}u^{b}={\hat{h}}_{ab}^{\left(1\right)}u^{b}=\ldots =0$, we will simplify the analysis by restricting all of these objects to be conformal to $h_{ab}$, i.e.

(22) $$\begin{array}{cc} {\bar{h}}_{ab}^{\left(1\right)} & ={\bar{H}}^{\left(1\right)}h_{ab}\;,\;{\bar{H}}^{\left(1\right)}\equiv \frac{1}{3}h^{ab}{\bar{h}}_{ab}^{\left(1\right)}=\frac{1}{3}{\bar{g}}^{AB}g_{AB}\;,\\ {\bar{h}}_{ab}^{\left(2\right)} & ={\bar{H}}^{\left(2\right)}h_{ab}\;,\;{\bar{H}}^{\left(2\right)}\equiv \frac{1}{3}h^{ab}{\bar{h}}_{ab}^{\left(2\right)}=\frac{1}{3}g^{AB}{\bar{g}}_{AB}\;,\\ {\hat{h}}_{ab}^{\left(1\right)} & ={\hat{H}}^{\left(1\right)}h_{ab}\;,\;{\hat{H}}^{\left(1\right)}\equiv \frac{1}{3}h^{ab}{\hat{h}}_{ab}^{\left(1\right)}=\frac{1}{3}{\hat{g}}^{AB}g_{AB}=\frac{1}{3}{\bar{M}}_{A}^{A}\;,\\ {\hat{h}}_{ab}^{\left(2\right)} & ={\hat{H}}^{\left(2\right)}h_{ab}\;,\;{\hat{H}}^{\left(2\right)}\equiv \frac{1}{3}h^{ab}{\hat{h}}_{ab}^{\left(2\right)}=\frac{1}{3}g^{AB}{\hat{g}}_{AB}=\frac{1}{3}M_{A}^{A}\;. \end{array}$$

## 4. The Nuts and Bolts of the Action Variation

We will now show that the proper-time derivatives ${\dot{g}}^{AB}$, ${\dot{\bar{g}}}^{AB}$, and ${\dot{\hat{g}}}^{AB}$ are directly connected to $\sigma_{ab}$, Θ, and $h_{ab}$. The implication of this is that any recovery of, say, the Navier–Stokes equations via the action principle means that ${\dot{g}}^{AB}$, ${\dot{\bar{g}}}^{AB}$, and ${\dot{\hat{g}}}^{AB}$ must be included as independent variables in the field variations.

The result follows because the master function Λ is commonly left unspecified in the action-based approach: usually, only its existence and the fields/fluxes it depends on are postulated. If an explicit master function can be provided, then the dependence of this on the fields’ derivatives will automatically be taken care of by the variational principle. We also note that [29] works around this issue by considering the dissipative fluxes as functionals of, say, the “metric” $g_{AB}$. In the present context, however, we try to avoid that as this would inevitably make the discussion somewhat phenomenological.

### 4.1. Matter Space Maps and Metric Derivatives

In Appendix A.3, it is shown (in Equation (A28)) that

(23) $$u^{a}=\frac{1}{3!}\epsilon^{bcda}\Psi_{b}^{B}\Psi_{c}^{C}\Psi_{d}^{D}\epsilon_{BCD}=\frac{1}{3!}\epsilon^{bcda}{\bar{\Psi }}_{b}^{B}{\bar{\Psi }}_{c}^{C}{\bar{\Psi }}_{d}^{D}{\bar{\epsilon }}_{BCD}\;.$$

This leads to the important consistency check that

(24) $$u^{a}\Psi_{a}^{A}=u^{a}\nabla_{a}X^{A}=L_{u}X^{A}=0\;⟹\;u^{a}{\bar{\Psi }}_{a}^{A}=L_{u}{\bar{X}}^{A}={\bar{M}}_{B}^{A}\left(u^{a}\Psi_{a}^{B}\right)=0\;,$$

which must hold because the map $\Psi_{a}^{A}$ is contracted four times on $\epsilon^{bcda}$ but $X^{A}$ has only three components. This means the $X^{A}$ and ${\bar{X}}^{A}$ are Lie-dragged along the fluid worldlines, which is expected because the basic role of the maps $\Psi_{a}^{A}$ and ${\bar{\Psi }}_{a}^{A}$ is to identify specific wordlines on spacetime with specific points in the matter spaces.

Because ${\bar{f}}^{A}$ is a function of $X^{A}$, then ${\bar{M}}_{B}^{A}$ is also a function of $X^{A}$, and because $f^{A}$ is a function of ${\bar{X}}^{A}$, then $M_{B}^{A}$ is a function of ${\bar{X}}^{A}$. Given that $L_{u}X^{A}=0=L_{u}{\bar{X}}^{A}$, we see

(25) $$L_{u}{\bar{M}}_{B}^{A}=0=L_{u}M_{B}^{A}\;.$$

Once the maps are specified at a given point on a worldline, they will not change on future points of the same worldline, which is ultimately due to our assumption that the particle and entropy spaces are diffeomorphic to each other.

To establish rules for taking derivatives of the matter space metrics, we need to develop further properties of the maps $\Psi_{a}^{A}$ and ${\bar{\Psi }}_{a}^{A}$: First, because the $X^{A}$ are scalars, then

(26) $$\nabla_{b}\Psi_{a}^{A}=\nabla_{a}\Psi_{b}^{A}\;.$$

This and the Lie dragging of the $X^{A}$ along $u^{a}$ allows us to write

(27) $$u^{b}\nabla_{b}\Psi_{a}^{A}=u^{b}\nabla_{a}\Psi_{b}^{A}=-\Psi_{b}^{A}\nabla_{a}u^{b}\;.$$

Hence, the Lie derivative of $\Psi_{a}^{A}$ with respect to $u^{a}$ is

(28) $$L_{u}\Psi_{a}^{A}=u^{b}\nabla_{b}\Psi_{a}^{A}+\Psi_{b}^{A}\nabla_{a}u^{b}=-\Psi_{b}^{A}\nabla_{a}u^{b}+\Psi_{b}^{A}\nabla_{a}u^{b}=0\;,$$

and similarly

(29) $$L_{u}{\bar{\Psi }}_{a}^{A}=0\;;$$

therefore, the maps are also Lie dragged along the worldlines. These can be combined to show

(30) $$\begin{array}{cc} u^{c}\nabla_{c}\left(\Psi_{a}^{A}\Psi_{b}^{B}\right) & =-\left(\Psi_{c}^{A}\Psi_{b}^{B}\nabla_{a}u^{c}+\Psi_{a}^{A}\Psi_{c}^{B}\nabla_{b}u^{c}\right)\;. \end{array}$$

Using Equation (28), we see that

(31) $$\begin{array}{ccc} {\dot{g}}^{AB} & \equiv & L_{u}\left(\Psi_{a}^{A}\Psi_{b}^{B}g^{ab}\right)\\ & = & \Psi_{a}^{A}\Psi_{b}^{B}L_{u}g^{ab}\\ & = & -2\Psi_{a}^{A}\Psi_{b}^{B}{⊥}_{c}^{a}{⊥}_{d}^{b}\nabla^{(c}u^{d)}\\ & = & -2\Psi_{a}^{A}\Psi_{b}^{B}\left(\sigma^{ab}+\frac{1}{3}\Theta h^{ab}\right)\;, \end{array}$$

where ${⊥}_{b}^{a}=h_{b}^{a}$. We also have

(32) $$\begin{array}{cc} {\dot{\bar{g}}}^{AB} & \equiv L_{u}\left({\bar{\Psi }}_{a}^{A}{\bar{\Psi }}_{b}^{B}g^{ab}\right)={\bar{M}}_{C}^{A}{\bar{M}}_{D}^{B}{\dot{g}}^{CD}\;, \end{array}$$

and

(33) $$\begin{array}{cc} {\dot{\hat{g}}}^{AB} & \equiv L_{u}\left(\Psi_{a}^{A}{\bar{\Psi }}_{b}^{B}g^{ab}\right)={\bar{M}}_{C}^{B}{\dot{g}}^{AC}\;. \end{array}$$

If we contract both sides of Equation (31) with $g^{AB}$, we have

(34) $$\begin{array}{cc} g_{AB}{\dot{g}}^{AB} & =-2\Theta ={\bar{g}}_{AB}{\dot{\bar{g}}}^{AB}={\hat{g}}_{AB}{\dot{\hat{g}}}^{AB}\;. \end{array}$$

Later, when we take partial derivatives of Equation (79) as one of the necessary steps of the action principle, the three quantities $g^{AB}$, ${\bar{g}}^{AB}$, and ${\hat{g}}^{AB}$ are treated as being independent. This prompts us to introduce

(35) $$\begin{array}{cc} \Theta^{\left(1\right)} & =-\frac{1}{2}{\bar{g}}_{AB}{\dot{\bar{g}}}^{AB}\;,\\ \Theta^{\left(2\right)} & =-\frac{1}{2}g_{AB}{\dot{g}}^{AB}\;,\\ \Theta^{\left(3\right)} & =-\frac{1}{2}{\hat{g}}_{AB}{\dot{\hat{g}}}^{AB}\;, \end{array}$$

to recognize the independence of $g^{AB}$, ${\bar{g}}^{AB}$, and ${\hat{g}}^{AB}$. In the variations that occur in the action, we need to recognize also that the three $\Theta^{\left(i\right)}$ are independent of each other. Once the variations are completed, then the three $\Theta^{\left(i\right)}$ can be set equal to each other (as in (34)).

The conformal factors ${\hat{H}}^{\left(1\right)}$ and ${\hat{H}}^{\left(2\right)}$ satisfy ${\dot{\hat{H}}}^{\left(i\right)}=0$ (i=1,2) since the first is a function only of $X^{A}$ and the second depends on only ${\bar{X}}^{A}$. The proper-time derivative ${\dot{\bar{H}}}^{\left(i\right)}$ is more complicated, namely,

(36) $$\begin{array}{cc} {\dot{\bar{H}}}^{\left(i\right)} & =\frac{1}{3}\left({\dot{\bar{g}}}^{AB}g_{AB}+{\bar{g}}^{AB}{\dot{g}}_{AB}\right)\\ \; & =-\frac{2}{3}\left[\left({\bar{\Psi }}_{a}^{A}{\bar{\Psi }}_{b}^{B}g_{AB}\right)-h^{cd}\left({\bar{\Psi }}_{c}^{A}\Psi_{a}^{C}g_{AC}\right)\left({\bar{\Psi }}_{d}^{B}\Psi_{b}^{D}g_{BD}\right)\right]\left(\sigma^{ab}+\frac{1}{3}\Theta h^{ab}\right)\\ \; & =-\frac{2}{3}\left[{\bar{H}}^{\left(i\right)}-{\left({\hat{H}}^{\left(i\right)}\right)}^{2}\right]h_{ab}\left(\sigma^{ab}+\frac{1}{3}\Theta h^{ab}\right)\\ \; & =\frac{2}{3}\left[{\bar{H}}^{\left(i\right)}-{\left({\hat{H}}^{\left(i\right)}\right)}^{2}\right]\frac{d\left(\ln n\right)}{d\tau }\;, \end{array}$$

where we have used the fact that because $\nabla_{a}n^{a}=0$ we can replace Θ with

(37) $$\Theta =-\frac{d\left(\ln n\right)}{d\tau }\;.$$

This implies that if ${\bar{H}}^{\left(i\right)}\left(\tau \right)=0$ for every value τ, and $n^{2/3}\left(\tau \right)$ does not remain constant, then ${\hat{H}}^{\left(i\right)}=0$.

Finally, we will work out the proper-time derivative of $\epsilon^{ABC}$. Begin by noting that

(38) $$\begin{array}{cc} u^{a}\nabla_{a}\det\left[g^{DE}\right] & =u^{a}\nabla_{a}\left(\frac{1}{3!}{\left[ABC\right]}_{D}{\left[DEF\right]}_{D}g^{AD}g^{BE}g^{CF}\right)\\ \; & =\frac{1}{2}{\left[CAB\right]}_{D}{\left[FDE\right]}_{D}g^{AD}g^{BE}{\dot{g}}^{CF}\\ \; & =\det\left[g^{DE}\right]g_{CF}{\dot{g}}^{CF}\\ \; & =-2\det\left[g^{DE}\right]\Theta \;, \end{array}$$

and therefore

(39) $${\dot{\epsilon }}^{ABC}=-\epsilon^{ABC}\Theta \;,\;{\dot{\bar{\epsilon }}}^{ABC}=-{\bar{\epsilon }}^{ABC}\Theta \;.$$

### 4.2. The Lagrangian Displacement

The key step to finding the correct equations of motion is to make sure that the variations $\delta n^{a}$ and $\delta s^{a}$ incorporate the Lie dragging of $X^{A}$ and ${\bar{X}}^{A}$. We achieve this by using the Lagrangian displacement $\Delta =\delta +L_{\xi }$, where $L_{\xi }$ is the Lie derivative along a spacetime displacement $\xi^{a}$. It is a measure of how a quantity changes with respect to fluid observers, who ride along with the worldlines. When we consider the action principle, we are then looking for variations $\delta X^{A}$ that lead to $\delta S_{F}=0$.

When a worldline is varied it must still be the case that its own $X^{A}$ and ${\bar{X}}^{A}$ remain fixed. The implication, then, is that $\delta X^{A}$ and $\xi^{a}$ must be such that they lead to $\Delta X^{A}=0$; hence, we find

(40) $$\begin{array}{cc} \delta X^{A} & =-L_{\xi }X^{A}=-\xi^{a}\partial_{a}X^{A}=-\Psi_{a}^{A}\xi^{a}\;,\\ \delta {\bar{X}}^{A} & ={\bar{M}}_{B}^{A}\delta X^{B}=-{\bar{M}}_{B}^{A}\Psi_{a}^{B}\xi^{a}=-{\bar{\Psi }}_{a}^{A}\xi^{a}\;. \end{array}$$

Obviously,

(41) $$\Delta {\bar{M}}_{B}^{A}=\Delta M_{B}^{A}=0\;.$$

The next thing is to use these to “fix” the variations $\delta n^{a}$ and $\delta s^{a}$ so that the action principle delivers viable equations of motion and an energy–momentum–stress tensor that can be inserted into the Einstein equations to determine the gravitational field.

We will start by deriving $\Delta g^{AB}$, $\Delta {\bar{g}}^{AB}$, and $\Delta {\hat{g}}^{AB}$. To facilitate this, we can show

(42) $$\Delta {\bar{\Psi }}_{a}^{A}=0\;.$$

Now, we find for $\Delta g^{AB}$, $\Delta {\bar{g}}^{AB}$, and $\Delta {\hat{g}}^{AB}$ that

(43) $$\begin{array}{cc} \Delta g^{AB} & =\Delta \left(\Psi_{a}^{A}\Psi_{b}^{B}g^{ab}\right)\\ \; & =\Psi_{a}^{A}\Psi_{b}^{B}\Delta g^{ab}\\ \; & =\Psi_{a}^{A}\Psi_{b}^{B}\left[\delta g^{ab}-2\nabla^{(a}\xi^{b)}\right]\;,\\ \Delta {\bar{g}}^{AB} & ={\bar{M}}_{C}^{A}{\bar{M}}_{D}^{B}\Delta g^{CD}\;,\\ \Delta {\hat{g}}^{AB} & ={\bar{M}}_{C}^{B}\Delta g^{AC}\;, \end{array}$$

where we have used the essential relation

(44) $$\Delta g^{ab}=\delta g^{ab}-2\nabla^{(a}\xi^{b)}\;.$$

It will be the case that we need to incorporate ${\dot{g}}^{AB}=u^{a}\nabla_{a}g^{AB}$ into our scheme, meaning we will have to also work out $\Delta {\dot{g}}^{AB}$. The starting point is

(45) $$\Delta {\dot{g}}^{AB}=\left(\partial_{a}g^{AB}\right)\Delta u^{a}+u^{a}\Delta \left(\partial_{a}g^{AB}\right)\;.$$

From Equation (A28) in Appendix A.3, we can infer that

(46) $$\Delta u^{a}=-\frac{1}{2}\left(u_{b}u_{c}\Delta g^{bc}\right)u^{a}\;,$$

where we have used

(47) $$\Delta \epsilon^{abcd}=\frac{1}{2}\epsilon^{abcd}g_{ef}\Delta g^{ef}\;,\;\Delta \epsilon_{ABC}=-\frac{1}{2}\epsilon_{ABC}g_{DE}\Delta g^{DE}\;.$$

Next (see (A29) in Appendix A.4 for details),

(48) $$\begin{array}{cc} \Delta \left(\partial_{a}g^{AB}\right) & =\partial_{a}\left(\Psi_{b}^{A}\Psi_{c}^{B}\Delta g^{bc}\right)\;, \end{array}$$

and therefore (see Equation (A30) in Appendix A.4),

(49) $$\begin{array}{cc} \Delta {\dot{g}}^{AB} & =\Psi_{a}^{A}\Psi_{b}^{B}\left\{{⊥}_{c}^{(a}{⊥}_{d}^{b)}u^{e}\nabla_{e}\left(\Delta g^{cd}\right)+\left[{⊥}_{e}^{(a}{⊥}_{f}^{b)}\left(\nabla^{(e}u^{f)}\right)u_{c}u_{d}-2{⊥}_{c}^{(a}{⊥}_{e}^{b)}\nabla_{d}u^{e}\right]\Delta g^{cd}\right\}\;, \end{array}$$

(50) $$\begin{array}{cc} \Delta {\dot{\bar{g}}}^{AB} & ={\bar{M}}_{C}^{A}{\bar{M}}_{D}^{B}\Delta {\dot{g}}^{CD}\;, \end{array}$$

(51) $$\begin{array}{cc} \Delta {\dot{\hat{g}}}^{AB} & ={\bar{M}}_{C}^{B}\Delta {\dot{g}}^{AC}\;. \end{array}$$

## 5. The Field Equations

The “trick” that incorporates dissipation in the variational formulation is to specify that the functional dependencies of $n_{ABC}$ and $s_{ABC}$ are

(52) $$n_{ABC}=n_{ABC}\left(X^{D}\right)\;,\;s_{ABC}=s_{ABC}\left({\bar{X}}^{D},{\bar{g}}^{DE},g^{DE},{\hat{g}}^{DE},{\dot{\bar{g}}}^{DE},{\dot{g}}^{DE},{\dot{\hat{g}}}^{DE}\right)\;.$$

It is clear that $\Delta n_{ABC}=0$, since it only depends on $X^{A}$. Consequently, the particle flux creation rate $\Gamma_{n}$ is shown to vanish; i.e., using the fact that $\nabla_{[a}\Psi_{b]}^{B}=0$, etc., we have

(53) $$\begin{array}{cc} \nabla_{a}n^{a} & =\frac{1}{3!}\epsilon^{bcda}\nabla_{[a}n_{bcd]}\\ \; & =\frac{1}{3!}\epsilon^{bcda}\nabla_{[a}\left(\Psi_{b}^{B}\Psi_{c}^{C}\Psi_{d]}^{D}n_{BCD}\right)\\ \; & =-\frac{1}{3!}\epsilon^{bcda}\Psi_{[b}^{B}\Psi_{c}^{C}\Psi_{d}^{D}\Psi_{a]}^{A}\frac{\partial n_{BCD}}{\partial X^{A}}\equiv 0\;. \end{array}$$

However, the extra dependencies for $s_{ABC}$, as we will see below, lead to a non-zero entropy creation $\Gamma_{s}=\nabla_{a}s^{a}$.

### 5.1. Construction of $\delta n^{a}$

To work out $\delta n^{a}$, we first determine $\Delta n_{ABC}$, using the form given in Equation (52):

(54) $$\begin{array}{cc} \Delta n_{ABC} & =\frac{\partial n_{ABC}}{\partial X^{D}}\Delta X^{D}=0\;. \end{array}$$

Since $\Delta n_{ABC}=0$ and $\Delta \Psi_{a}^{A}=0$, we see $\Delta n_{abc}=0$ and therefore

(55) $$\delta n_{abc}=-L_{\xi }n_{abc}\;.$$

Noting that

(56) $$\begin{array}{cc} \frac{1}{3!}\epsilon^{bcda}L_{\xi }n_{bcd} & =\xi^{b}\nabla_{b}n^{a}-\left(n^{d}\nabla_{d}\xi^{a}-n^{a}\nabla_{d}\xi^{d}\right)\;, \end{array}$$

we see

(57) $$\begin{array}{cc} \delta n^{a} & =-\left[\xi^{b}\nabla_{b}n^{a}-\left(n^{d}\nabla_{d}\xi^{a}-n^{a}\nabla_{d}\xi^{d}\right)\right]+\frac{1}{2}n^{a}g_{bc}\delta g^{bc}\;, \end{array}$$

where we have used

(58) $$\delta \epsilon^{bcda}=\frac{1}{2}\epsilon^{bcda}g_{ef}\delta g^{ef}\;.$$

Finally, we have

(59) $$\begin{array}{cc} \mu_{a}\delta n^{a} & =\left(-2n^{b}\nabla_{[b}\mu_{a]}\right)\xi^{a}+\frac{1}{2}\mu_{a}n^{a}g_{bc}\delta g^{bc}+\nabla_{b}\left(\mu_{a}n^{b}\xi^{a}-\mu_{a}n^{a}\xi^{b}\right)\;. \end{array}$$

### 5.2. Construction of $\delta s^{a}$

To perform the setup for $\delta s^{a}$, we note that $\Delta s_{ABC}$ is

(60) $$\begin{array}{cc} \Delta s_{ABC} & =\frac{\partial s_{ABC}}{\partial {\bar{g}}^{DE}}\Delta {\bar{g}}^{DE}+\frac{\partial s_{ABC}}{\partial g^{DE}}\Delta g^{DE}+\frac{\partial s_{ABC}}{\partial {\hat{g}}^{DE}}\Delta {\hat{g}}^{DE}\\ \; & +\frac{\partial s_{ABC}}{\partial {\dot{\bar{g}}}^{DE}}\Delta {\dot{\bar{g}}}^{DE}+\frac{\partial s_{ABC}}{\partial {\dot{g}}^{DE}}\Delta {\dot{g}}^{DE}+\frac{\partial s_{ABC}}{\partial {\dot{\hat{g}}}^{DE}}\Delta {\dot{\hat{g}}}^{DE}\;, \end{array}$$

where the form given in Equation (52) has been used. Recalling that $\Delta {\bar{\Psi }}_{a}^{A}=0$, we see

(61) $$\Delta s_{abc}={\bar{\Psi }}_{a}^{[A}{\bar{\Psi }}_{b}^{B}{\bar{\Psi }}_{c}^{C]}\Delta s_{ABC}\;,$$

which implies

(62) $$\delta s_{abc}=-L_{\xi }s_{abc}+{\bar{\Psi }}_{a}^{[A}{\bar{\Psi }}_{b}^{B}{\bar{\Psi }}_{c}^{C]}\Delta s_{ABC}\;.$$

Now we can rewrite $\delta s^{a}$ as

(63) $$\begin{array}{cc} \delta s^{a} & =-\frac{1}{3!}\epsilon^{bcda}L_{\xi }s_{bcd}+\frac{1}{3!}\epsilon^{bcda}{\bar{\Psi }}_{[b}^{A}{\bar{\Psi }}_{c}^{B}{\bar{\Psi }}_{d]}^{C}\Delta s_{ABC}+\frac{1}{2}s^{a}g_{bc}\delta g^{bc}\\ \; & =-\left[\xi^{b}\nabla_{b}s^{a}-\left(s^{d}\nabla_{d}\xi^{a}-s^{a}\nabla_{d}\xi^{d}\right)\right]+\left(\frac{1}{3!}\epsilon^{bcda}{\bar{\Psi }}_{[b}^{A}{\bar{\Psi }}_{c}^{B}{\bar{\Psi }}_{d]}^{C}{\bar{\epsilon }}_{ABC}\right)\left(\frac{1}{3!}{\bar{\epsilon }}^{DEF}\Delta s_{DEF}\right)\\ \; & +\frac{1}{2}s^{a}g_{bc}\delta g^{bc}\\ \; & =-\left[\xi^{b}\nabla_{b}s^{a}-\left(s^{d}\nabla_{d}\xi^{a}-s^{a}\nabla_{d}\xi^{d}\right)\right]+\left(\frac{1}{3!}{\bar{\epsilon }}^{ABC}\Delta s_{ABC}\right)u^{a}+\frac{1}{2}s^{a}g_{bc}\delta g^{bc}\;, \end{array}$$

so that

(64) $$\begin{array}{cc} \Theta_{a}\delta s^{a} & =-\left(2s^{b}\nabla_{[b}\Theta_{a]}+\Gamma_{s}\Theta_{a}\right)\xi^{a}-\frac{1}{3!}\Theta^{BCD}\Delta s_{BCD}-\frac{1}{2}Tsg_{ab}\delta g^{ab}\\ \; & +\nabla_{b}\left[\left(\Theta_{a}s^{a}\delta_{c}^{b}-\Theta_{c}s^{b}\right)\xi^{c}\right]\;. \end{array}$$

When we define, following the notation in [23],

(65) $$\begin{array}{cc} {\bar{D}}_{ab} & =\frac{1}{3}\Theta^{ABC}\frac{\partial s_{ABC}}{\partial {\bar{g}}^{DE}}{\bar{\Psi }}_{a}^{D}{\bar{\Psi }}_{b}^{E}\;,\;D_{ab}=\frac{1}{3}\Theta^{ABC}\frac{\partial s_{ABC}}{\partial g^{DE}}\Psi_{a}^{D}\Psi_{b}^{E}\;,\;{\hat{D}}_{ab}=\frac{1}{3}\Theta^{ABC}\frac{\partial s_{ABC}}{\partial {\hat{g}}^{DE}}\Psi_{a}^{D}{\bar{\Psi }}_{b}^{E}\;,\\ {\bar{D}}_{ab} & =\frac{1}{3}\Theta^{ABC}\frac{\partial s_{ABC}}{\partial {\dot{\bar{g}}}^{DE}}{\bar{\Psi }}_{a}^{D}{\bar{\Psi }}_{b}^{E}\;,\;D_{ab}=\frac{1}{3}\Theta^{ABC}\frac{\partial s_{ABC}}{\partial {\dot{g}}^{DE}}\Psi_{a}^{D}\Psi_{b}^{E}\;,\;{\hat{D}}_{ab}=\frac{1}{3}\Theta^{ABC}\frac{\partial s_{ABC}}{\partial {\dot{\hat{g}}}^{DE}}\Psi_{a}^{D}{\bar{\Psi }}_{b}^{E}\;, \end{array}$$

(where ${\bar{D}}_{ab}={\bar{D}}_{ba}$, $u^{b}{\bar{D}}_{ab}=u^{b}{\bar{D}}_{ba}=0$, and likewise for the others), and

(66) $$\begin{array}{cc} D_{ab}^{T} & ={\bar{D}}_{ab}+D_{ab}+{\hat{D}}_{ab}\;,\\ D_{ab}^{T} & ={\bar{D}}_{ab}+D_{ab}+{\hat{D}}_{ab}\;, \end{array}$$

we find (see (A31) in Appendix A.5)

(67) $$\begin{array}{cc} \frac{1}{3!}\Theta^{BCD}\Delta s_{BCD} & =\nabla^{b}\left[D_{ba}^{T}+D_{cd}^{T}\left(\nabla^{(c}u^{d)}\right)u_{b}u_{a}-2D_{c(a}^{T}\nabla_{b)}u^{c}-\nabla_{c}\left(D_{ba}^{T}u^{c}\right)\right]\xi^{a}\\ \; & +\frac{1}{2}\left[D_{ab}^{T}+D_{cd}^{T}\left(\nabla^{(c}u^{d)}\right)u_{a}u_{b}-2D_{c(a}^{T}\nabla_{b)}u^{c}-\nabla_{c}\left(D_{ab}^{T}u^{c}\right)\right]\delta g^{ab}\\ \; & -\nabla^{a}\left\{\left[D_{ab}^{T}+D_{cd}^{T}\left(\nabla^{(c}u^{d)}\right)u_{a}u_{b}-2D_{c(a}^{T}\nabla_{b)}u^{c}-\nabla_{c}\left(D_{ab}^{T}u^{c}\right)\right]\xi^{b}\right\}\\ \; & +\nabla_{c}\left[\frac{1}{2}D_{ab}^{T}u^{c}\left(\delta g^{ab}-2\nabla^{(a}\xi^{b)}\right)\right]\;. \end{array}$$

Therefore,

(68) $$\begin{array}{cc} \Theta_{a}\delta s^{a} & =-\left\{2s^{b}\nabla_{[b}\Theta_{a]}+\Gamma_{s}\Theta_{a}+\nabla^{b}\left[D_{ba}^{T}+D_{cd}^{T}\left(\nabla^{(c}u^{d)}\right)u_{b}u_{a}-2D_{c(a}^{T}\nabla_{b)}u^{c}-\nabla_{c}\left(D_{ba}^{T}u^{c}\right)\right]\right\}\xi^{a}\\ \; & -\frac{1}{2}\left[Tsg_{ab}+D_{ab}^{T}+D_{cd}^{T}\left(\nabla^{(c}u^{d)}\right)u_{a}u_{b}-2D_{c(a}^{T}\nabla_{b)}u^{c}-\nabla_{c}\left(D_{ab}^{T}u^{c}\right)\right]\delta g^{ab}\\ \; & +\nabla^{a}\left\{\left[D_{ab}^{T}+D_{cd}^{T}\left(\nabla^{(c}u^{d)}\right)u_{a}u_{b}-2D_{c(a}^{T}\nabla_{b)}u^{c}-\nabla_{c}\left(D_{ab}^{T}u^{c}\right)\right]\xi^{b}\right\}\\ \; & -\nabla_{c}\left[\frac{1}{2}D_{ab}^{T}u^{c}\left(\delta g^{ab}-2\nabla^{(a}\xi^{b)}\right)\right]+\nabla_{b}\left[\left(\Theta_{a}s^{a}\delta_{c}^{b}-\Theta_{c}s^{b}\right)\xi^{c}\right]\;. \end{array}$$

### 5.3. The General Variation of the Action

Now that both $\delta n^{a}$ and $\delta s^{a}$ are in place, we find that the variation of the action is

(69) $$\begin{array}{cc} \delta S_{F} & =\int_{M}d^{4}x\sqrt{-g}\left\{-\left(2n^{b}\nabla_{[b}\mu_{a]}+2s^{b}\nabla_{[b}\Theta_{a]}+\Gamma_{s}\Theta_{a}+\nabla^{b}\left[D_{ba}^{T}+D_{cd}^{T}\left(\nabla^{(c}u^{d)}\right)u_{b}u_{a}\right.\right.\right.\\ \; & \left.\left.\left.-2D_{c(a}^{T}\nabla_{b)}u^{c}-\nabla_{c}\left(D_{ba}^{T}u^{c}\right)\right]\right)\xi^{a}+\frac{1}{2}\left[\left(\Lambda +\mu n+Ts\right)g^{ab}+\mu^{a}n^{b}\right.\right.\\ \; & \left.+\Theta^{a}s^{b}+\left.D_{T}^{ab}+D_{cd}^{T}\left(\nabla^{(c}u^{d)}\right)u^{a}u^{b}-2{D_{T}^{(a}}_{\left|c\right|}\nabla^{b)}u^{c}-\nabla_{c}\left(D_{T}^{ab}u^{c}\right)\right]\delta g_{ab}\right\}\\ \; & +B.T.\;, \end{array}$$

where B.T. represents all the “boundary terms” that come from the total derivatives. The equation of motion is

(70) $$\begin{array}{cc} 0 & =2n^{b}\nabla_{[b}\mu_{a]}+2s^{b}\nabla_{[b}\Theta_{a]}+\Gamma_{s}\Theta_{a}+\nabla^{b}\left[D_{ba}^{T}+D_{cd}^{T}\left(\nabla^{(c}u^{d)}\right)u_{b}u_{a}\right.\\ \; & \left.-2D_{c(a}^{T}\nabla_{b)}u^{c}-\nabla_{c}\left(D_{ba}^{T}u^{c}\right)\right]\;, \end{array}$$

the entropy creation rate is (see Equation (A32) in Appendix A.5)

(71) $$\begin{array}{cc} T\Gamma_{s} & =-D_{ba}^{T}u^{c}\nabla_{c}\left[\nabla^{(b}u^{a)}\right]-\left[D_{ba}^{T}-2D_{c(a}^{T}\nabla_{b)}u^{c}\right]\nabla^{(b}u^{a)}\;, \end{array}$$

and the energy–momentum–stress tensor is

(72) $$\begin{array}{cc} T^{ab} & =\Psi g^{ab}+\left(\Psi -\Lambda \right)u^{a}u^{b}+D_{T}^{ab}\\ \; & +D_{cd}^{T}\left(\nabla^{(c}u^{d)}\right)u^{a}u^{b}-2{D_{T}^{(a}}_{\left|c\right|}\nabla^{b)}u^{c}-\nabla_{c}\left(D_{T}^{ab}u^{c}\right)\;, \end{array}$$

with the generalized pressure Ψ defined as

(73) $$\Psi =\Lambda -\mu_{a}n^{a}-\Theta_{a}s^{a}=\Lambda +\mu n+Ts\;.$$

## 6. A Navier–Stokes(-ish) Model

As a direct application of the formal developments, we consider a specific model for the functional dependence of $s_{ABC}$. As a precursor, we look more closely at the generic form of the entropy creation rate derived above, by inserting the decomposition of $\nabla_{a}u_{b}$ given in Equation (19) into Equation (71). We then find

(74) $$\begin{array}{cc} T\Gamma_{s} & =-D_{ab}^{T}\left(\sigma^{ab}+\frac{1}{3}\Theta h^{ab}\right)-\frac{1}{3}D_{ab}^{T}\left(\dot{\Theta }+\frac{2}{3}\Theta^{2}\right)h^{ab}\\ \; & -D_{ab}^{T}\left[{\dot{\sigma }}^{ab}-2\left({\sigma_{c}}^{a}+{\varpi_{c}}^{a}+\frac{2}{3}\Theta \delta_{c}^{a}\right)\sigma^{bc}\right]\;. \end{array}$$

This is useful because we can use the Onsager technique (in this context, see [30]) of identifying appropriate thermodynamic “forces” and “fluxes” in order to ensure that the second law of thermodynamics is respected: $\Gamma_{s}\geq 0$. In this example, one finds that the following gives the usual Navier–Stokes entropy creation rate, but a different equation of motion and energy–momentum–stress tensor, namely, the choice

(75) $$\begin{array}{cc} D_{ab}^{T} & =-T\left\{\left(\eta -2\lambda \right)\sigma_{ab}+\left[\left(\zeta +\frac{1}{3}\eta \right)+\lambda \left(\frac{2}{3}+\frac{1}{\Theta }\frac{d\left(\ln\Theta \right)}{d\tau }\right)\right]\Theta h_{ab}\right\}\;,\\ D_{ab}^{T} & =T\lambda h_{ab}\;, \end{array}$$

leads to

(76) $$\Gamma_{s}=\eta \sigma_{ab}\sigma^{ab}+\left(\zeta +\frac{1}{3}\eta \right)\Theta^{2}\;.$$

The corresponding equation of motion is

(77) $$\begin{array}{cc} 0 & =2n^{b}\nabla_{[b}\mu_{a]}+2s^{b}\nabla_{[b}\Theta_{a]}+\Gamma_{s}\Theta_{a}-\nabla^{b}\left\{T\left[\eta \sigma_{ab}+\left(\zeta +\frac{1}{3}\eta \right)\Theta h_{ab}\right]\right.\\ \; & \left.+T\lambda \left[\frac{7}{3}+\frac{1}{\Theta }u^{c}\nabla_{c}\ln\left(T\lambda \Theta \right)\right]\Theta h_{ab}\right\}\;, \end{array}$$

and the energy–momentum–stress tensor is

(78) $$\begin{array}{cc} T^{ab} & =\Psi g^{ab}+\left(\Psi -\Lambda \right)u^{a}u^{b}-T\left[\eta \sigma^{ab}+\left(\zeta +\frac{1}{3}\eta \right)\Theta h^{ab}\right]\\ \; & -T\lambda \left[\frac{7}{3}+\frac{1}{\Theta }u^{c}\nabla_{c}\ln\left(T\lambda \Theta \right)\right]\Theta h^{ab}\;. \end{array}$$

The Onsager construction is well grounded in both experimental and theoretical chemistry (for example, when considering systems with many reaction rates [31]) and the same is the case for physics applications. But this is not all that we are seeking here; for example, in the Onsager strategy the coefficients η, ζ, and λ are determined “externally” assuming that the system has experienced some (linear) deviation away from some prescribed equilibrium. In contrast, the variational derivation involved no notion of equilibrium with everything determined by the action principle.

As a proof of principle and demonstration of how the calculation should proceed, we will consider a specific form for the entropy density and then push through the formulae given above for the equation of motion, entropy creation rate, and energy–momentum–stress tensor. We will find that the natural matter and entropy space elements of such a construct (${\bar{g}}^{AB}$, ${\dot{\bar{g}}}^{AB}$, etc.) have built-in properties for the otherwise arbitrary coefficients that are used to tie them together in $s_{ABC}$.

We now consider a specific form for $s_{ABC}$, which has only linear terms in ${\dot{\bar{g}}}^{AB}$ and ${\dot{g}}^{AB}$. Specifically, we start from

(79) $$s_{ABC}=s_{ABC}^{\left(0\right)}+\sum_{j=1}^{2}\sum_{i=1}^{2}{\bar{s}}_{ABC}^{\left(i,j\right)}{\bar{H}}^{\left(i\right)}\Theta^{\left(j\right)}+{\bar{s}}_{ABC}^{\left(1\right)}{\dot{g}}^{DE}{\bar{g}}_{DE}+s_{ABC}^{\left(1\right)}{\dot{\bar{g}}}^{DE}g_{DE}\;.$$

All of the “$\bar{s}$”, “*s*”, and “$\hat{s}$” coefficients are functions of only ${\bar{X}}^{A}$. From this, we can construct the entropy density:

(80) $$\begin{array}{cc} s & =\frac{1}{3!}{\bar{\epsilon }}^{ABC}s_{ABC}\\ \; & =s^{\left(0\right)}+\sum_{j=1}^{2}\sum_{i=1}^{2}{\bar{s}}^{\left(i,j\right)}{\bar{H}}^{\left(i\right)}\Theta^{\left(j\right)}+{\bar{s}}^{\left(1\right)}{\dot{g}}^{DE}{\bar{g}}_{DE}+s^{\left(1\right)}{\dot{\bar{g}}}^{DE}g_{DE}\;. \end{array}$$

where

(81) $$s^{\left(0\right)}=\frac{1}{3!}{\bar{\epsilon }}^{ABC}s_{ABC}^{\left(0\right)}\;,\;{\bar{s}}^{\left(i,j\right)}=\frac{1}{3!}{\bar{\epsilon }}^{ABC}{\bar{s}}_{ABC}^{\left(i,j\right)}\;,\;{\bar{s}}^{\left(1\right)}=\frac{1}{3!}{\bar{\epsilon }}^{ABC}{\bar{s}}_{ABC}^{\left(1\right)}\;,\;s^{\left(1\right)}=\frac{1}{3!}{\bar{\epsilon }}^{ABC}s_{ABC}^{\left(1\right)}\;.$$

Since ${\dot{\bar{X}}}^{D}=0$, and using Equation (39), we see

(82) $${\dot{s}}^{\left(0\right)}=-s^{\left(0\right)}\Theta \;,\;{\dot{\bar{s}}}^{\left(i,j\right)}=-{\bar{s}}^{\left(i,j\right)}\Theta \;,\;{\dot{\bar{s}}}^{\left(1\right)}=-{\bar{s}}^{\left(1\right)}\Theta \;,\;{\dot{s}}^{\left(1\right)}=-s^{\left(1\right)}\Theta \;,$$

or

(83) $$\nabla_{a}\left(s^{\left(0\right)}u^{a}\right)=0\;,\;\nabla_{a}\left({\bar{s}}^{\left(i,j\right)}u^{a}\right)=0\;,\;\nabla_{a}\left({\bar{s}}^{\left(1\right)}u^{a}\right)=0\;,\;\nabla_{a}\left(s^{\left(1\right)}u^{a}\right)=0\;.$$

Using the various derivatives given in Equation (A33) in Appendix A.6, we can show that

(84) $$\begin{array}{cc} \frac{\partial s_{ABC}}{\partial {\bar{g}}^{DE}} & =\frac{1}{3}\sum_{j=1}^{2}\left[{\bar{s}}_{ABC}^{\left(1,j\right)}g_{DE}-\frac{1}{2}{\bar{s}}_{ABC}^{\left(2,j\right)}g^{FG}\left({\bar{g}}_{FD}{\bar{g}}_{GE}+{\bar{g}}_{GD}{\bar{g}}_{FE}\right)\right]\Theta \\ \; & +\frac{1}{2}\sum_{i=1}^{2}{\bar{s}}_{ABC}^{\left(i,1\right)}{\bar{H}}^{\left(i\right)}{\bar{g}}_{FD}{\bar{g}}_{GE}{\dot{\bar{g}}}^{FG}-\frac{1}{2}{\bar{s}}_{ABC}^{\left(1\right)}{\dot{g}}^{FG}\left({\bar{g}}_{FD}{\bar{g}}_{GE}+{\bar{g}}_{GD}{\bar{g}}_{FE}\right)\;,\\ \frac{\partial s_{ABC}}{\partial g^{DE}} & =\frac{1}{3}\sum_{j=1}^{2}\left[{\bar{s}}_{ABC}^{\left(2,j\right)}{\bar{g}}_{DE}-\frac{1}{2}{\bar{s}}_{ABC}^{\left(1,j\right)}{\bar{g}}^{FG}\left(g_{FD}g_{GE}+g_{GD}g_{FE}\right)\right]\Theta \\ \; & +\frac{1}{2}\sum_{i=1}^{2}{\bar{s}}_{ABC}^{\left(i,2\right)}{\bar{H}}^{\left(i\right)}g_{FD}g_{GE}{\dot{g}}^{FG}-\frac{1}{2}s_{ABC}^{\left(1\right)}{\dot{\bar{g}}}^{FG}\left(g_{FD}g_{GE}+g_{GD}g_{FE}\right)\;,\\ \frac{\partial s_{ABC}}{\partial {\dot{\bar{g}}}^{DE}} & =\left(-\frac{1}{2}\sum_{i=1}^{2}{\bar{s}}_{ABC}^{\left(i,1\right)}{\bar{H}}^{\left(i\right)}{\bar{g}}_{DE}+s_{ABC}^{\left(1\right)}g_{DE}\right)\;,\\ \frac{\partial s_{ABC}}{\partial {\dot{g}}^{DE}} & =\left(-\frac{1}{2}\sum_{i=1}^{2}{\bar{s}}_{ABC}^{\left(i,2\right)}{\bar{H}}^{\left(i\right)}g_{DE}+{\bar{s}}_{ABC}^{\left(1\right)}{\bar{g}}_{DE}\right)\;. \end{array}$$

The four tensors ${\bar{D}}_{ab}$, $D_{ab}$, ${\bar{D}}_{ab}$, and $D_{ab}$ are, respectively,

(85) $$\begin{array}{cc} {\bar{D}}_{ab} & =\frac{1}{3}\Theta^{ABC}\frac{\partial s_{ABC}}{\partial {\bar{g}}^{DE}}{\bar{\Psi }}_{a}^{D}{\bar{\Psi }}_{b}^{E}\\ \; & =-2T\left\{\left[{\bar{s}}^{\left(1,1\right)}{\bar{H}}^{\left(1\right)}+{\bar{s}}^{\left(2,1\right)}{\bar{H}}^{\left(2\right)}-2{\bar{s}}^{\left(1\right)}{\left({\hat{H}}^{\left(2\right)}\right)}^{2}\right]\sigma_{ab}\right.\\ \; & \left.+\frac{1}{3}\left[{\bar{s}}^{\left(2,1\right)}{\bar{H}}^{\left(2\right)}-{\bar{s}}^{\left(1,2\right)}{\bar{H}}^{\left(1\right)}+\left({\bar{s}}^{\left(2,1\right)}+{\bar{s}}^{\left(2,2\right)}-2{\bar{s}}^{\left(1\right)}\right){\left({\hat{H}}^{\left(2\right)}\right)}^{2}\right]\Theta h_{ab}\right\}\;, \end{array}$$

(86) $$\begin{array}{cc} D_{ab} & =\frac{1}{3}\Theta^{ABC}\frac{\partial s_{ABC}}{\partial g^{DE}}\Psi_{a}^{D}\Psi_{b}^{E}\\ \; & =-2T\left\{\left[{\bar{s}}^{\left(1,2\right)}{\bar{H}}^{\left(1\right)}+{\bar{s}}^{\left(2,2\right)}{\bar{H}}^{\left(2\right)}-2s^{\left(1\right)}{\left({\hat{H}}^{\left(1\right)}\right)}^{2}\right]\sigma_{ab}\right.\\ \; & \left.+\frac{1}{3}\left[{\bar{s}}^{\left(1,2\right)}{\bar{H}}^{\left(1\right)}-{\bar{s}}^{\left(2,1\right)}{\bar{H}}^{\left(2\right)}+\left({\bar{s}}^{\left(1,1\right)}+{\bar{s}}^{\left(1,2\right)}-2s^{\left(1\right)}\right){\left({\hat{H}}^{\left(1\right)}\right)}^{2}\right]\Theta h_{ab}\right\}\;, \end{array}$$

(87) $$\begin{array}{cc} {\bar{D}}_{ab} & =\frac{1}{3}\Theta^{ABC}\frac{\partial s_{ABC}}{\partial {\dot{\bar{g}}}^{DE}}{\bar{\Psi }}_{a}^{D}{\bar{\Psi }}_{b}^{E}\\ \; & =-2T\left(\frac{1}{2}\sum_{i=1}^{2}{\bar{s}}^{\left(i,1\right)}{\bar{H}}^{\left(i\right)}-s^{\left(1\right)}{\bar{H}}^{\left(1\right)}\right)h_{ab}\;, \end{array}$$

(88) $$\begin{array}{cc} D_{ab} & =\frac{1}{3}\Theta^{ABC}\frac{\partial s_{ABC}}{\partial {\dot{g}}^{DE}}\Psi_{a}^{D}\Psi_{b}^{E}\\ \; & =-2T\left(\frac{1}{2}\sum_{i=1}^{2}{\bar{s}}^{\left(i,2\right)}{\bar{H}}^{\left(i\right)}-{\bar{s}}^{\left(1\right)}{\bar{H}}^{\left(2\right)}\right)h_{ab}\;. \end{array}$$

Finally, the two “dissipation” tensors $D_{ab}^{T}$ and $D_{ab}^{T}$ are, respectively,

(89) $$\begin{array}{cc} D_{ab}^{T} & =-2T\left\{\left[\sum_{i=1}^{2}\left({\bar{s}}^{\left(i,1\right)}+{\bar{s}}^{\left(i,2\right)}\right){\bar{H}}^{\left(i\right)}-2{\bar{s}}^{\left(1\right)}{\left({\hat{H}}^{\left(2\right)}\right)}^{2}-2s^{\left(1\right)}{\left({\hat{H}}^{\left(1\right)}\right)}^{2}\right]\sigma_{ab}\right.\\ \; & \left.+\frac{1}{3}\left[\sum_{i=1}^{2}\left({\bar{s}}^{\left(i,1\right)}+{\bar{s}}^{\left(i,2\right)}\right){\left({\hat{H}}^{\left(i\right)}\right)}^{2}-2s^{\left(1\right)}{\left({\hat{H}}^{\left(1\right)}\right)}^{2}-2{\bar{s}}^{\left(1\right)}{\left({\hat{H}}^{\left(2\right)}\right)}^{2}\right]\Theta h_{ab}\right\}\\ \; & =Tc_{1}\sigma_{ab}+Tc_{2}\Theta h_{ab}\;,\\ D_{ab}^{T} & =-2T\left[\frac{1}{2}\sum_{i=1}^{2}\left({\bar{s}}^{\left(i,1\right)}+{\bar{s}}^{\left(i,2\right)}\right){\bar{H}}^{\left(i\right)}-\left({\bar{s}}^{\left(1\right)}{\bar{H}}^{\left(2\right)}+s^{\left(1\right)}{\bar{H}}^{\left(1\right)}\right)\right]h_{ab}\\ \; & =Tc_{3}h_{ab}\;, \end{array}$$

where

(90) $$\begin{array}{cc} c_{1} & =-2\sum_{i=1}^{2}\left({\bar{s}}^{\left(i,1\right)}+{\bar{s}}^{\left(i,2\right)}\right){\bar{H}}^{\left(i\right)}+4{\bar{s}}^{\left(1\right)}{\left({\hat{H}}^{\left(2\right)}\right)}^{2}+4s^{\left(1\right)}{\left({\hat{H}}^{\left(1\right)}\right)}^{2}\;,\\ c_{2} & =-\frac{2}{3}\left[\sum_{i=1}^{2}\left({\bar{s}}^{\left(i,1\right)}+{\bar{s}}^{\left(i,2\right)}\right){\left({\hat{H}}^{\left(i\right)}\right)}^{2}-2s^{\left(1\right)}{\left({\hat{H}}^{\left(1\right)}\right)}^{2}-2{\bar{s}}^{\left(1\right)}{\left({\hat{H}}^{\left(2\right)}\right)}^{2}\right]\;,\\ c_{3} & =-\sum_{i=1}^{2}\left({\bar{s}}^{\left(i,1\right)}+{\bar{s}}^{\left(i,2\right)}\right){\bar{H}}^{\left(i\right)}+2\left({\bar{s}}^{\left(1\right)}{\bar{H}}^{\left(2\right)}+s^{\left(1\right)}{\bar{H}}^{\left(1\right)}\right)\;. \end{array}$$

Note that the coefficients $c_{i}$ satisfy the following system of linear, first-order differential equations:

(91) $$\begin{array}{cc} {\dot{c}}_{1} & =\left(2c_{2}-\frac{5}{3}c_{1}\right)\Theta \;, \end{array}$$

(92) $$\begin{array}{cc} {\dot{c}}_{2} & =-c_{2}\Theta \;, \end{array}$$

(93) $$\begin{array}{cc} {\dot{c}}_{3} & =\left(c_{2}-\frac{5}{3}c_{3}\right)\Theta \;; \end{array}$$

therefore, keeping them static along fluid worldlines is not possible. This is a significant difference with the Onsager model given earlier at the start of this section where, in priniciple, its η, ζ, and λ coefficients satisfy (up to the choice of sign) no constraints or evolution equations.

The equation of motion is

(94) $$\begin{array}{cc} 0 & =2n^{b}\nabla_{[b}\mu_{a]}+2s^{b}\nabla_{[b}\Theta_{a]}+\Gamma_{s}\Theta_{a}+\nabla^{b}\left\{T\left[\left(c_{1}-2c_{3}\right)\sigma_{ab}+\left(c_{2}-\frac{2}{3}c_{3}\right)\Theta h_{ab}\right.\right.\\ \; & \left.\left.-c_{3}\Theta g_{ab}-\frac{1}{T}u^{c}\nabla_{c}\left(c_{3}T\right)h_{ab}\right]\right\}\;, \end{array}$$

while the entropy creation rate is determined to be

(95) $$\Gamma_{s}=\left(2c_{3}-c_{1}\right)\sigma_{ab}\sigma^{ab}+\left(\frac{2}{3}c_{3}-c_{2}\right)\Theta^{2}-c_{3}\dot{\Theta }\;,$$

and the energy–momentum–stress tensor is

(96) $$\begin{array}{cc} T^{ab} & =\left(\Psi -c_{3}\Theta \right)g^{ab}+\left(\Psi -\Lambda \right)u^{a}u^{b}+T\left[\left(c_{1}-2c_{3}\right)\sigma^{ab}+\left(c_{2}-\frac{2}{3}c_{3}\right)\Theta h^{ab}\right.\\ \; & \left.-\frac{1}{T}u^{c}\nabla_{c}\left(c_{3}T\right)h^{ab}\right]\;. \end{array}$$

The set of Equations (94)–(96) completes the dissipative fluid model that follows from the variational principle once we make the chosen simplifications and adopt the prescription in Equation (79). At this point, all that remains is to examine the results and decide if these equations are “acceptable” or not. A first hint of the latter follows from a comparison with (76) and (78). The equations we have arrived at clearly do not replicate the model built using Onsager-style reasoning. Of course, this was not our intention. We set out to develop an explicit model to illustrate the steps and assumptions required to go from $s_{ABC}$ to the final equation of motion, the entropy creation rate, and the energy–momentum–stress tensor. A notable feature of this model is that—unlike the Onsager approach or, indeed, every other state-of-the-art model for dissipative relativistic fluids—all functions and parameters (e.g., bulk and shear viscosity) are determined at the level of the action. In fact, even their evolutions along individual world lines are obtained within the formalism. This is conceptually important and there are valuable lessons to learn from the derivation. For example, it is evident that the bulk and shear viscosity should not be taken to be “constant” in a general nonlinear model. With a governing set of equations like (91)–(93), it is clear that the model must evolve with the flow. However, despite having some appealing features it is clear that the specific model we have arrived at is problematic. Most importantly, it is clear from (95) that the only way to ensure that the second law is enforced (locally) is to insist that $c_{3}$ vanishes at all times. This then leads to $c_{2}$ vanishing as well and we are left with a model having only $c_{1}\neq 0$, representing a system where the only dissipation channel is shear viscosity. This restricted model may have interesting applications, but it is clearly not the general model we were looking for. There is more work to do here.

## 7. Concluding Remarks

Building on the variational approach for dissipative relativistic fluids from [23], we have taken steps towards formulating an explicit action principle that connects with the familiar Navier–Stokes equations. In general, the variational approach is built around matter and entropy fluxes (taken to be the primary degrees of freedom) and dissipation arises if the dual three-form associated with a given flux is not closed. As discussed in [23], this allows us to represent a number of dissipative channels, but the general model is too “rich” to permit an intuitive interpretation. Given this, we introduced a number of simplifications aimed at reducing the complexity of the model and highlighting the key features. Most notably, we restricted ourselves to a single-fluid model. The motivation for this (somewhat drastic, given that we know that issues like heat/entropy flows require a multi-fluid approach [10]) assumption was to make contact with numerical simulations which tend to reduce the analysis to a single fluid for practical reasons.

Given the various simplifications introduced in our derivation of the fluid equations, the fact that the final result appears somewhat unfinished is perhaps not surprising. However, we would argue that the analysis provides several useful lessons. For example, we have seen that the proper time derivative of the matter space “metric” must be included in the matter Lagrangian in order to recover the expected terms associated with bulk and shear viscosity. The discussion also shows that evolution equations along fluid worldlines arise naturally in the model, a feature one might expect from a relativistic description. At the same time, the construction added a less desirable term to the entropy creation rate. The upshot is that the final model presented here is satisfactory—in the sense that it is compatible with the second law (implemented locally)—as long as we only allow for the presence of shear viscosity. The addition of bulk viscosity requires further thought.

To make progress, we may go back to the beginning and relax the simplifying assumptions one by one. This will make the discussion more involved, but at this point this seems unavoidable. Noting that, from an implementation point of view, single-fluid models are much easier to work with than multi-fluid ones, it would certainly be interesting to see how much closer to a “workable” dissipative fluid model we can get without relaxing the single-fluid assumption. If we have to account for the explicit multi-fluid aspects, then the framework for this already exists (see [10]), but we need to be mindful of the fact that we are still quite far from having developed such models to the level where they are ready for numerical implementation.

## Data Availability

Data is contained within the article.

## Appendix A. Details Behind the Derivation of Important Relations

### Appendix A.1. Metric and Map Inverses

Note that $\epsilon^{abcd}$ is an essential component of this process of pull-back and push-forward. It and its inverse $\epsilon_{abcd}$ satisfy some useful identities:

(A1) $$\begin{array}{cc} \epsilon^{abcd}\epsilon_{efgh} & =-4!\delta_{e}^{[a}\delta_{f}^{b}\delta_{g}^{c}\delta_{h}^{d]}\;,\\ \epsilon^{abcd}\epsilon_{afgh} & =-3!\delta_{f}^{[b}\delta_{g}^{c}\delta_{h}^{d]}\;,\\ \epsilon^{abcd}\epsilon_{abgh} & =-2{!}^{2}\delta_{g}^{[c}\delta_{h}^{d]}\;,\\ \epsilon^{abcd}\epsilon_{abch} & =-3!\delta_{h}^{d}\;,\\ \epsilon^{abcd}\epsilon_{abcd} & =-4!\;. \end{array}$$

In a similar way, we can introduce the three-forms $\left\{\epsilon^{ABC},{\bar{\epsilon }}^{ABC}\right\}$ and inverses $\left\{\epsilon_{ABC},{\bar{\epsilon }}_{ABC}\right\}$, which have a similar set of identities:

(A2) $$\begin{array}{cc} \epsilon^{ABC}\epsilon_{DEF} & ={\bar{\epsilon }}^{ABC}{\bar{\epsilon }}_{DEF}=3!\delta_{D}^{[A}\delta_{E}^{B}\delta_{F}^{C]}\;, \end{array}$$

(A3) $$\begin{array}{cc} \epsilon^{ABC}\epsilon_{AEF} & ={\bar{\epsilon }}^{ABC}{\bar{\epsilon }}_{AEF}=2\delta_{E}^{[B}\delta_{F}^{C]}\;, \end{array}$$

(A4) $$\begin{array}{cc} \epsilon^{ABC}\epsilon_{ABF} & ={\bar{\epsilon }}^{ABC}{\bar{\epsilon }}_{ABF}=2\delta_{F}^{C}\;, \end{array}$$

(A5) $$\begin{array}{cc} \epsilon^{ABC}\epsilon_{ABC} & ={\bar{\epsilon }}^{ABC}{\bar{\epsilon }}_{ABC}=3!\;. \end{array}$$

Using basic linear algebra techniques (Cramer’s rule), it can be shown that the matter space metric inverses $g_{AB}$, ${\bar{g}}_{AB}$, ${\hat{g}}_{AB}$ are given by

(A6) $$\begin{array}{cc} g^{AC}g_{CB} & =\delta_{B}^{A}\;⟹\;g_{AB}=\frac{1}{2}\epsilon_{ACE}\epsilon_{BDF}g^{CD}g^{EF}\;,\\ {\bar{g}}^{AC}{\bar{g}}_{CB} & =\delta_{B}^{A}\;⟹\;{\bar{g}}_{AB}=\frac{1}{2}{\bar{\epsilon }}_{ACE}{\bar{\epsilon }}_{BDF}{\bar{g}}^{CD}{\bar{g}}^{EF}\;,\\ {\hat{g}}^{AC}{\hat{g}}_{CB} & =\delta_{B}^{A}\;⟹\;{\hat{g}}_{AB}=\frac{1}{2}{\hat{\epsilon }}_{ACE}{\hat{\epsilon }}_{BDF}{\hat{g}}^{CD}{\hat{g}}^{EF}\;, \end{array}$$

where

(A7) $$\begin{array}{cc} \epsilon^{ABC} & =\sqrt{\det\left[g^{DE}\right]}{\left[ABC\right]}^{U}\;,\;\epsilon_{ABC}=\frac{1}{\sqrt{\det\left[g^{DE}\right]}}{\left[ABC\right]}_{D}\;,\\ {\bar{\epsilon }}^{ABC} & =\sqrt{\det\left[{\bar{g}}^{DE}\right]}{\left[ABC\right]}^{U}\;,\;{\bar{\epsilon }}_{ABC}=\frac{1}{\sqrt{\det\left[{\bar{g}}^{DE}\right]}}{\left[ABC\right]}_{D}\;,\\ {\hat{\epsilon }}^{ABC} & =\sqrt{\det\left[{\hat{g}}^{DE}\right]}{\left[ABC\right]}^{U}\;,\;{\hat{\epsilon }}_{ABC}=\frac{1}{\sqrt{\det\left[{\hat{g}}^{DE}\right]}}{\left[ABC\right]}_{D}\;, \end{array}$$

(A8) $$\begin{array}{c} {\left[ABC\right]}^{U}=3!\delta_{1}^{[A}\delta_{2}^{B}\delta_{3}^{C]}\;,\;{\left[ABC\right]}_{D}=3!\delta_{[A}^{1}\delta_{B}^{2}\delta_{C]}^{3}\;, \end{array}$$

and

(A9) $$\begin{array}{cc} \det\left[g^{DE}\right] & =\frac{1}{3!}{\left[ABC\right]}_{D}{\left[DEF\right]}_{D}g^{AD}g^{BE}g^{CF}\;,\\ \det\left[{\bar{g}}^{DE}\right] & =\frac{1}{3!}{\left[ABC\right]}_{D}{\left[DEF\right]}_{D}{\bar{g}}^{AD}{\bar{g}}^{BE}{\bar{g}}^{CF}\;,\\ \det\left[{\hat{g}}^{DE}\right] & =\frac{1}{3!}{\left[ABC\right]}_{D}{\left[DEF\right]}_{D}{\hat{g}}^{AD}{\hat{g}}^{BE}{\hat{g}}^{CF}\;. \end{array}$$

Because of Equation (A2),

(A10) $${\left[ABC\right]}^{U}{\left[DEF\right]}_{D}=3!\delta_{D}^{[A}\delta_{E}^{B}\delta_{F}^{C]}\;.$$

It is also the case that

(A11) $${\bar{M}}_{B}^{A}=\frac{1}{2}\epsilon_{M}^{ACE}\epsilon_{BDF}^{M}M_{C}^{D}M_{E}^{F}\;,$$

where

(A12) $$\epsilon_{M}^{ABC}=\sqrt{\det\left[M_{E}^{D}\right]}{\left[ABC\right]}^{U}\;,\;\epsilon_{ABC}^{M}=\frac{1}{\sqrt{\det\left[M_{E}^{D}\right]}}{\left[ABC\right]}_{D}\;,$$

and

(A13) $$\det\left[M_{E}^{D}\right]=\frac{1}{3!}{\left[ABC\right]}_{D}{\left[DEF\right]}_{U}M_{D}^{A}M_{E}^{B}M_{F}^{C}\;.$$

### Appendix A.2. Mappings between $g^{AB}$, ${\bar{g}}^{AB}$, and ${\hat{g}}^{AB}$

In order to establish the rule for mapping $g_{AB}$ to ${\bar{g}}_{AB}$, and vice versa, we can show that the standard rules involving Jacobians apply. For this, we work out $\det\left[g^{DE}\right]$, and the rest follow similarly:

(A14) $$\begin{array}{cc} \det\left[g^{DE}\right] & =\frac{1}{3!}{\left[ABC\right]}_{D}{\left[DEF\right]}_{D}g^{AD}g^{BE}g^{CF}\\ \; & =\frac{1}{3!}{\left[ABC\right]}_{D}{\left[DEF\right]}_{D}M_{G}^{A}M_{J}^{D}{\bar{g}}^{GJ}M_{H}^{B}M_{K}^{E}{\bar{g}}^{HK}M_{I}^{C}M_{L}^{F}{\bar{g}}^{IL}\\ \; & =\frac{1}{3!}{\left[ABC\right]}_{D}M_{G}^{A}M_{H}^{B}M_{I}^{C}{\left[DEF\right]}_{D}M_{J}^{D}M_{K}^{E}M_{L}^{F}{\bar{g}}^{GJ}{\bar{g}}^{HK}{\bar{g}}^{IL}\\ \; & =\frac{1}{3!}{\left[ABC\right]}_{D}M_{G}^{A}M_{H}^{B}M_{I}^{C}{\left[DEF\right]}_{D}M_{J}^{D}M_{K}^{E}M_{L}^{F}\delta_{M}^{[G}\delta_{N}^{H}\delta_{O}^{I]}\delta_{P}^{[J}\delta_{Q}^{K}\delta_{R}^{L]}{\bar{g}}^{MP}{\bar{g}}^{NQ}{\bar{g}}^{OR}\\ \; & =\left(\frac{1}{3!}{\left[GHI\right]}^{U}{\left[ABC\right]}_{D}M_{G}^{A}M_{H}^{B}M_{I}^{C}\right)\left(\frac{1}{3!}{\left[JKL\right]}^{U}{\left[DEF\right]}_{D}M_{J}^{D}M_{K}^{E}M_{L}^{F}\right)\\ \; & \left(\frac{1}{3!}{\left[MNO\right]}_{D}{\left[PQR\right]}_{D}{\bar{g}}^{MP}{\bar{g}}^{NQ}{\bar{g}}^{OR}\right)\\ \; & ={\left(\det\left[M_{B}^{A}\right]\right)}^{2}\det\left[{\bar{g}}^{DE}\right]\;. \end{array}$$

This means that the inverse $g_{AB}$ is mapped to ${\bar{g}}_{AB}$ via

(A15) $$\begin{array}{cc} M_{A}^{C}M_{B}^{D}g_{CD} & =\frac{1}{2}M_{A}^{C}M_{B}^{D}\epsilon_{CEG}\epsilon_{DFH}M_{I}^{E}M_{K}^{F}{\bar{g}}^{IK}M_{J}^{G}M_{L}^{H}{\bar{g}}^{JL}={\bar{g}}_{AB}\;; \end{array}$$

that is,

(A16) $${\bar{g}}_{AB}=M_{A}^{C}M_{B}^{D}g_{CD}\;,\;g_{AB}={\bar{M}}_{A}^{C}{\bar{M}}_{B}^{D}{\bar{g}}_{CD}\;.$$

Starting with the fact that $\det\left[\delta_{B}^{A}\right]=1$, and using Equation (6), it can be shown that

(A17) $$\det\left[{\bar{M}}_{B}^{A}\right]=1/\det\left[M_{B}^{A}\right]\;.$$

Finally, we also determine the connections with $\det\left[{\hat{g}}^{DE}\right]$:

(A18) $$\begin{array}{cc} \det\left[{\hat{g}}^{DE}\right] & =\det\left[{\bar{M}}_{B}^{A}\right]\det\left[g^{AB}\right]\;. \end{array}$$

With this, it can be shown that

(A19) $$g_{AB}={\bar{M}}_{B}^{C}{\hat{g}}_{AC}\;,\;{\hat{g}}_{AB}=M_{B}^{C}g_{AC}\;.$$

Consequently,

(A20) $$\begin{array}{cc} g^{AB}{\hat{g}}_{AB} & =g^{AB}M_{B}^{C}g_{AC}=\left(g^{BA}g_{AC}\right)M_{B}^{C}=M_{A}^{A}\;,\\ {\hat{g}}^{AB}g_{AB} & =g^{AC}{\bar{M}}_{C}^{B}g_{AB}=\left(g^{AC}g_{AB}\right){\bar{M}}_{C}^{B}={\bar{M}}_{A}^{A}\;. \end{array}$$

### Appendix A.3. Matter Space Volume Forms

The next step is to establish the rules for identifying the spacetime objects with their matter space counterparts, and to determine how to connect the particle space objects with the entropy space ones. Two essential ingredients for this are the completely antisymmetric objects $\epsilon_{ABC}$ and ${\bar{\epsilon }}_{ABC}$, whose defining properties are given in Equation (A5).

It must be the case that $n_{ABC}$ and $s_{ABC}$ are proportional to $\epsilon_{ABC}$ and ${\bar{\epsilon }}_{ABC}$, respectively, i.e., $n_{ABC}=N\epsilon_{ABC}$ and $s_{ABC}=S{\bar{\epsilon }}_{ABC}$, and that $\mu^{ABC}$ and $\Theta^{ABC}$ are proportional to $\epsilon^{ABC}$ and ${\bar{\epsilon }}^{ABC}$, respectively, i.e., $\mu^{ABC}=M\epsilon^{ABC}$ and $\Theta^{ABC}=T{\bar{\epsilon }}^{ABC}$. Equation (A5) then implies

(A21) $$\begin{array}{cc} N & =\frac{1}{3!}\epsilon^{ABC}n_{ABC}\;,\;S=\frac{1}{3!}{\bar{\epsilon }}^{ABC}s_{ABC}\;,\\ M & =\frac{1}{3!}\epsilon_{ABC}\mu^{ABC}\;,\;T=\frac{1}{3!}{\bar{\epsilon }}_{ABC}\Theta^{ABC}\;. \end{array}$$

It is easy to see that

(A22) $$\epsilon_{abc}\equiv u^{d}\epsilon_{dabc}=\Psi_{a}^{A}\Psi_{b}^{B}\Psi_{c}^{C}n_{ABC}/n={\bar{\Psi }}_{a}^{A}{\bar{\Psi }}_{b}^{B}{\bar{\Psi }}_{c}^{C}s_{ABC}/s\;,$$

and

(A23) $$\begin{array}{cc} \Psi_{a}^{A}\Psi_{b}^{B}\Psi_{c}^{C}\epsilon^{abc} & \equiv \Psi_{a}^{A}\Psi_{b}^{B}\Psi_{c}^{C}u^{d}{\epsilon^{abc}}_{d}=\mu^{ABC}/\mu \;,\\ {\bar{\Psi }}_{a}^{A}{\bar{\Psi }}_{b}^{B}{\bar{\Psi }}_{c}^{C}\epsilon^{abc} & \equiv {\bar{\Psi }}_{a}^{A}{\bar{\Psi }}_{b}^{B}{\bar{\Psi }}_{c}^{C}u^{d}{\epsilon^{abc}}_{d}=\Theta^{ABC}/T\;. \end{array}$$

From the definitions of $\mu_{abc}$ and $\Theta_{abc}$ we have

(A24) $$\begin{array}{cc} M & =\frac{1}{3!}\epsilon_{ABC}\mu^{ABC}=\mu \left(\frac{1}{3!}\epsilon^{abc}\epsilon_{ABC}\Psi_{a}^{A}\Psi_{b}^{B}\Psi_{c}^{C}\right)=\mu \left(\frac{1}{3!}\epsilon^{abc}\epsilon_{abc}\right)=\mu \;,\\ T & =\frac{1}{3!}{\bar{\epsilon }}_{ABC}\Theta^{ABC}=T\left(\frac{1}{3!}\epsilon^{abc}{\bar{\epsilon }}_{ABC}{\bar{\Psi }}_{a}^{A}{\bar{\Psi }}_{b}^{B}{\bar{\Psi }}_{c}^{C}\right)=T\left(\frac{1}{3!}\epsilon^{abc}\epsilon_{abc}\right)=T\;. \end{array}$$

This implies

(A25) $$\begin{array}{cc} \epsilon^{ABC} & =\mu^{ABC}/\mu =\Psi_{a}^{A}\Psi_{b}^{B}\Psi_{c}^{C}\mu^{abc}/\mu =\Psi_{a}^{A}\Psi_{b}^{B}\Psi_{c}^{C}\epsilon^{abc}\;,\\ {\bar{\epsilon }}^{ABC} & =\Theta^{ABC}/T={\bar{\Psi }}_{a}^{A}{\bar{\Psi }}_{b}^{B}{\bar{\Psi }}_{c}^{C}\Theta^{abc}/T={\bar{\Psi }}_{a}^{A}{\bar{\Psi }}_{b}^{B}{\bar{\Psi }}_{c}^{C}\epsilon^{abc}\;, \end{array}$$

and therefore N=n and S=s. It is now straightforward to show that

(A26) $${\bar{\epsilon }}^{ABC}={\bar{M}}_{D}^{A}{\bar{M}}_{E}^{B}{\bar{M}}_{F}^{C}\epsilon^{DEF}\;,\;\epsilon^{ABC}=M_{D}^{A}M_{E}^{B}M_{F}^{C}{\bar{\epsilon }}^{DEF}\;,$$

and similarly for ${\bar{\epsilon }}_{ABC}$ and $\epsilon_{ABC}$. Finally, we find

(A27) $$\epsilon_{abc}=\Psi_{a}^{D}\Psi_{b}^{E}\Psi_{c}^{F}\epsilon_{DEF}={\bar{\Psi }}_{a}^{A}{\bar{\Psi }}_{b}^{B}{\bar{\Psi }}_{c}^{C}{\bar{\epsilon }}_{ABC}\;,$$

and therefore

(A28) $$u^{a}=\frac{1}{3!}\epsilon^{bcda}\Psi_{b}^{B}\Psi_{c}^{C}\Psi_{d}^{D}\epsilon_{BCD}=\frac{1}{3!}\epsilon^{bcda}{\bar{\Psi }}_{b}^{B}{\bar{\Psi }}_{c}^{C}{\bar{\Psi }}_{d}^{D}{\bar{\epsilon }}_{BCD}\;.$$

### Appendix A.4. Matter Space Metric Variations

Steps leading to Equation (48) in the main text:

(A29) $$\begin{array}{cc} \Delta \left(\partial_{a}g^{AB}\right) & =\delta \left(\partial_{a}g^{AB}\right)+L_{\xi }\left(\partial_{a}g^{AB}\right)\\ \; & =\partial_{a}\left(\delta g^{AB}\right)+\xi^{b}\nabla_{b}\left(\partial_{a}g^{AB}\right)+\left(\partial_{b}g^{AB}\right)\nabla_{a}\xi^{b}\\ \; & =\partial_{a}\left(\delta g^{AB}\right)+\xi^{b}\nabla_{a}\left(\partial_{b}g^{AB}\right)+\left(\partial_{b}g^{AB}\right)\nabla_{a}\xi^{b}\\ \; & =\partial_{a}\left(\delta g^{AB}\right)+\partial_{a}\left(\xi^{b}\partial_{b}g^{AB}\right)\\ \; & =\partial_{a}\left(\delta g^{AB}+L_{\xi }g^{AB}\right)\\ \; & =\partial_{a}\left(\Delta g^{AB}\right)\\ \; & =\partial_{a}\left(\Psi_{b}^{A}\Psi_{c}^{B}\Delta g^{bc}\right)\;, \end{array}$$

The major steps used to develop the cross-listed Equation (49) in the main text:

(A30) $$\begin{array}{cc} \Delta {\dot{g}}^{AB} & =-{\dot{g}}^{AB}\left(\frac{1}{2}u_{b}u_{c}\Delta g^{bc}\right)+u^{a}\partial_{a}\left(\Psi_{b}^{A}\Psi_{c}^{B}{⊥}_{d}^{(b}{⊥}_{e}^{c)}\Delta g^{de}\right)\\ \; & =\Psi_{a}^{A}\Psi_{b}^{B}\nabla^{(a}u^{b)}\left(u_{c}u_{d}\Delta g^{cd}\right)\\ \; & -\left(\Psi_{a}^{A}\Psi_{b}^{B}+\Psi_{b}^{A}\Psi_{a}^{B}\right)\left(\nabla_{c}u^{a}\right){⊥}_{d}^{(b}{⊥}_{e}^{c)}\Delta g^{de}+\Psi_{a}^{A}\Psi_{b}^{B}u^{e}\nabla_{e}\left({⊥}_{c}^{(a}{⊥}_{d}^{b)}\Delta g^{cd}\right)\\ \; & =\Psi_{a}^{A}\Psi_{b}^{B}\nabla^{(a}u^{b)}\left(u_{c}u_{d}\Delta g^{cd}\right)\\ \; & -\Psi_{a}^{A}\Psi_{b}^{B}\left({⊥}_{d}^{b}\nabla_{c}u^{a}+{⊥}_{d}^{a}\nabla_{c}u^{b}\right){⊥}_{e}^{c}\Delta g^{de}+\Psi_{a}^{A}\Psi_{b}^{B}u^{e}\nabla_{e}\left({⊥}_{c}^{(a}{⊥}_{d}^{b)}\Delta g^{cd}\right)\\ \; & =\Psi_{a}^{A}\Psi_{b}^{B}\left[\nabla^{(a}u^{b)}\left(u_{c}u_{d}\Delta g^{cd}\right)-2\left({⊥}_{c}^{(a}\nabla_{e}u^{b)}\right){⊥}_{d}^{e}\Delta g^{cd}+u^{e}\nabla_{e}\left({⊥}_{c}^{(a}{⊥}_{d}^{b)}\Delta g^{cd}\right)\right]\\ \; & =\Psi_{a}^{A}\Psi_{b}^{B}\left\{\left[\left(\nabla^{(a}u^{b)}\right)u_{c}u_{d}-2{⊥}_{d}^{e}{⊥}_{c}^{(a}\nabla_{e}u^{b)}+u^{e}\nabla_{e}\left({⊥}_{c}^{(a}{⊥}_{d}^{b)}\right)\right]\Delta g^{cd}\right.\\ \; & \left.+{⊥}_{c}^{(a}{⊥}_{d}^{b)}u^{e}\nabla_{e}\left(\Delta g^{cd}\right)\right\}\\ \; & =\Psi_{a}^{A}\Psi_{b}^{B}\left\{{⊥}_{c}^{(a}{⊥}_{d}^{b)}u^{e}\nabla_{e}\left(\Delta g^{cd}\right)+\left[{⊥}_{e}^{(a}{⊥}_{f}^{b)}\left(\nabla^{(e}u^{f)}\right)u_{c}u_{d}\right.\right.\\ \; & \left.\left.-2{⊥}_{c}^{(a}{⊥}_{e}^{b)}\nabla_{d}u^{e}\right]\Delta g^{cd}\right\}\;. \end{array}$$

### Appendix A.5. Entropy Creation Rate Derivation

The major steps used to develop the cross-listed Equation (A31) in the main text:

(A31) $$\begin{array}{cc} \frac{1}{3!}\Theta^{BCD}\Delta s_{BCD} & =\frac{1}{2}D_{ab}^{T}\left[\delta g^{ab}-2\nabla^{(a}\xi^{b)}\right]+\frac{1}{2}D_{ab}^{T}\left\{{⊥}_{c}^{a}{⊥}_{d}^{b}u^{e}\nabla_{e}\left(\delta g^{cd}-2\nabla^{(c}\xi^{d)}\right)\right.\\ \; & \left.+\left[{⊥}_{(e}^{a}{⊥}_{f)}^{b}\left(\nabla^{e}u^{f}\right)u_{c}u_{d}-2{⊥}_{c}^{(a}{⊥}_{e}^{b)}\nabla_{d}u^{e}\right]\left(\delta g^{cd}-2\nabla^{(c}\xi^{d)}\right)\right\}\\ \; & =\frac{1}{2}D_{ab}^{T}\left[\delta g^{ab}-2\nabla^{(a}\xi^{b)}\right]+\frac{1}{2}\left\{D_{ab}^{T}u^{c}\nabla_{c}\left(\delta g^{ab}-2\nabla^{(a}\xi^{b)}\right)\right.\\ \; & \left.+\left[D_{cd}^{T}\left(\nabla^{(c}u^{d)}\right)u_{a}u_{b}-2D_{c(a}^{T}\nabla_{b)}u^{c}\right]\left(\delta g^{ab}-2\nabla^{(a}\xi^{b)}\right)\right\}\\ \; & =\frac{1}{2}\left[D_{ab}^{T}+D_{cd}^{T}\left(\nabla^{(c}u^{d)}\right)u_{a}u_{b}-2D_{c(a}^{T}\nabla_{b)}u^{c}-\nabla_{c}\left(D_{ab}^{T}u^{c}\right)\right]\\ \; & \left[\delta g^{ab}-2\nabla^{(a}\xi^{b)}\right]+\nabla_{c}\left[\frac{1}{2}D_{ab}^{T}u^{c}\left(\delta g^{ab}-2\nabla^{(a}\xi^{b)}\right)\right]\\ \; & =-\left[D_{ab}^{T}+D_{cd}^{T}\left(\nabla^{(c}u^{d)}\right)u_{a}u_{b}-2D_{c(a}^{T}\nabla_{b)}u^{c}-\nabla_{c}\left(D_{ab}^{T}u^{c}\right)\right]\nabla^{(a}\xi^{b)}\\ \; & +\frac{1}{2}\left[D_{ab}^{T}+D_{cd}^{T}\left(\nabla^{(c}u^{d)}\right)u_{a}u_{b}-2D_{c(a}^{T}\nabla_{b)}u^{c}-\nabla_{c}\left(D_{ab}^{T}u^{c}\right)\right]\delta g^{ab}\\ \; & +\nabla_{c}\left[\frac{1}{2}D_{ab}^{T}u^{c}\left(\delta g^{ab}-2\nabla^{(a}\xi^{b)}\right)\right]\\ \; & =\nabla^{b}\left[D_{ba}^{T}+D_{cd}^{T}\left(\nabla^{(c}u^{d)}\right)u_{b}u_{a}-2D_{c(a}^{T}\nabla_{b)}u^{c}-\nabla_{c}\left(D_{ba}^{T}u^{c}\right)\right]\xi^{a}\\ \; & +\frac{1}{2}\left[D_{ab}^{T}+D_{cd}^{T}\left(\nabla^{(c}u^{d)}\right)u_{a}u_{b}-2D_{c(a}^{T}\nabla_{b)}u^{c}-\nabla_{c}\left(D_{ab}^{T}u^{c}\right)\right]\delta g^{ab}\\ \; & -\nabla^{a}\left\{\left[D_{ab}^{T}+D_{cd}^{T}\left(\nabla^{(c}u^{d)}\right)u_{a}u_{b}-2D_{c(a}^{T}\nabla_{b)}u^{c}-\nabla_{c}\left(D_{ab}^{T}u^{c}\right)\right]\xi^{b}\right\}\\ \; & +\nabla_{c}\left[\frac{1}{2}D_{ab}^{T}u^{c}\left(\delta g^{ab}-2\nabla^{(a}\xi^{b)}\right)\right]\;, \end{array}$$

Steps leading to the entropy creation rate $\Gamma_{s}$ start with projecting the equation of motion Equation (70) onto $u^{a}$:

(A32) $$\begin{array}{cc} \left(-u^{a}\Theta_{a}\right)\Gamma_{s} & =u^{a}\nabla^{b}\left[D_{ba}^{T}+D_{cd}^{T}\left(\nabla^{(c}u^{d)}\right)u_{b}u_{a}-2D_{c(a}^{T}\nabla_{b)}u^{c}-\nabla_{c}\left(D_{ba}^{T}u^{c}\right)\right]\\ \; & =\nabla^{b}\left\{u^{a}\left[D_{ba}^{T}+D_{cd}^{T}\left(\nabla^{(c}u^{d)}\right)u_{b}u_{a}-2D_{c(a}^{T}\nabla_{b)}u^{c}-\nabla_{c}\left(D_{ba}^{T}u^{c}\right)\right]\right\}\\ \; & -\left[D_{ba}^{T}+D_{cd}^{T}\left(\nabla^{(c}u^{d)}\right)u_{b}u_{a}-2D_{c(a}^{T}\nabla_{b)}u^{c}-\nabla_{c}\left(D_{ba}^{T}u^{c}\right)\right]\nabla^{b}u^{a}\\ \; & =\nabla^{b}\left[D_{cd}^{T}\left(\nabla^{(c}u^{d)}\right)u^{a}u_{a}u_{b}-2u^{a}D_{c(a}^{T}\nabla_{b)}u^{c}-u^{a}\nabla_{c}\left(D_{ba}^{T}u^{c}\right)\right]\\ \; & -\left[D_{ba}^{T}-2D_{c(a}^{T}\nabla_{b)}u^{c}-\nabla_{c}\left(D_{ba}^{T}u^{c}\right)\right]\nabla^{b}u^{a}\\ \; & =\nabla^{b}\left[-D_{cd}^{T}\left(\nabla^{(c}u^{d)}\right)u_{b}-D_{cb}^{T}u^{a}\nabla_{a}u^{c}-\nabla_{c}\left(u^{a}D_{ba}^{T}u^{c}\right)+D_{ba}^{T}u^{c}\nabla_{c}u^{a}\right]\\ \; & -\left[D_{ba}^{T}-2D_{c(a}^{T}\nabla_{b)}u^{c}-\nabla_{c}\left(D_{ba}^{T}u^{c}\right)\right]\nabla^{(b}u^{a)}\\ \; & =-\nabla_{c}\left[D_{ba}^{T}u^{c}\left(\nabla^{(b}u^{a)}\right)\right]+\nabla_{c}\left(D_{ba}^{T}u^{c}\right)\nabla^{(b}u^{a)}-\left[D_{ba}^{T}-2D_{c(a}^{T}\nabla_{b)}u^{c}\right]\nabla^{(b}u^{a)}\\ \; & =-D_{ba}^{T}u^{c}\nabla_{c}\left[\nabla^{(b}u^{a)}\right]-\nabla_{c}\left(D_{ba}^{T}u^{c}\right)\nabla^{(b}u^{a)}+\nabla_{c}\left(D_{ba}^{T}u^{c}\right)\nabla^{(b}u^{a)}\\ \; & -\left[D_{ba}^{T}-2D_{c(a}^{T}\nabla_{b)}u^{c}\right]\nabla^{(b}u^{a)}\\ \; & =-D_{ba}^{T}u^{c}\nabla_{c}\left[\nabla^{(b}u^{a)}\right]-\left[D_{ba}^{T}-2D_{c(a}^{T}\nabla_{b)}u^{c}\right]\nabla^{(b}u^{a)}\;. \end{array}$$

### Appendix A.6. Useful Partial Derivatives

A few useful formulas are

(A33) $$\begin{array}{cc} \frac{\partial g_{AB}}{\partial g^{DE}} & =-\frac{1}{2}\left(g_{AD}g_{BE}+g_{AE}g_{BD}\right)\;,\\ \frac{\partial {\dot{g}}^{AB}}{\partial {\dot{g}}^{DE}} & =\frac{1}{2}\left(\delta_{D}^{A}\delta_{E}^{B}+\delta_{D}^{B}\delta_{E}^{A}\right)\;,\\ \frac{\partial \epsilon_{ABC}}{\partial g^{DE}} & =-\frac{1}{2}\epsilon_{ABC}\;g_{DE}\\ \frac{\partial \Theta }{\partial g^{DE}} & =-\frac{1}{2}\frac{\partial g_{AB}}{\partial g^{DE}}{\dot{g}}^{AB}=\frac{1}{2}g_{AD}g_{BE}{\dot{g}}^{AB}\\ \frac{\partial \Theta }{\partial {\dot{g}}^{DE}} & =-\frac{1}{2}\frac{\partial {\dot{g}}^{AB}}{\partial {\dot{g}}^{DE}}g_{AB}=-\frac{1}{2}g_{DE}\;,\\ \frac{\partial {\bar{H}}^{\left(1\right)}}{\partial {\bar{g}}^{DE}} & =\frac{1}{3}\frac{\partial {\bar{g}}^{AB}}{\partial {\bar{g}}^{DE}}g_{AB}=\frac{1}{3}g_{DE}\;,\\ \frac{\partial {\bar{H}}^{\left(1\right)}}{\partial g^{DE}} & =\frac{1}{3}\frac{\partial g_{AB}}{\partial g^{DE}}{\bar{g}}^{AB}=-\frac{1}{6}{\bar{g}}^{AB}\left(g_{AD}g_{BE}+g_{AE}g_{BD}\right)\;,\\ \frac{\partial {\bar{H}}^{\left(2\right)}}{\partial g^{DE}} & =\frac{1}{3}\frac{\partial g^{AB}}{\partial g^{DE}}{\bar{g}}_{AB}=\frac{1}{3}{\bar{g}}_{DE}\;,\\ \frac{\partial {\bar{H}}^{\left(2\right)}}{\partial {\bar{g}}^{DE}} & =\frac{1}{3}\frac{\partial {\bar{g}}_{AB}}{\partial {\bar{g}}^{DE}}g^{AB}=-\frac{1}{6}g^{AB}\left({\bar{g}}_{AD}{\bar{g}}_{BE}+{\bar{g}}_{AE}{\bar{g}}_{BD}\right)\;. \end{array}$$
